# Heterometallic Extended Metal Atom Chain Complexes with Carboxylate (O_2_CX) Axial Ligands

**DOI:** 10.1021/acs.inorgchem.6c02287

**Published:** 2026-08-10

**Authors:** Amelia M. Wheaton, Milton Acosta, John F. Berry

**Affiliations:** Department of Chemistry, 5228 University of Wisconsin − Madison, 1101 University Ave., Madison, Wisconsin 53706, United States

## Abstract

This work reports the preparation of Mo_2_Ni­(dpa)_4_(O_2_CC_6_H_4_X)_2_ heterometallic extended metal atom chain complexes with a systematic series of substituted benzoate axial ligands (dpa = 2,2′-dipyridylamido). Here, X is the *para* substituent: CH_3_ (**1_CH**_**3**_), H (**1_H**), I (**1_I**), CN (**1_CN**), or CF_3_ (**1_CF**_**3**_). The synthetic route used to prepare these compounds builds upon our previous work on axial ligand substitutions with pseudohalides, as all compounds in series **1** are prepared in useful yields via the combination of the previously reported Mo_2_Ni­(dpa)_4_(OTf)_2_ precursor with two equivalents of a [NEt_4_]­[O_2_CC_6_H_4_X] carboxylate salt. Crystallographic, spectroscopic, and magnetic measurements demonstrate that the compounds in series **1** possess similar structural and spectroscopic features and support an *S* = 1 Ni^2+^ paramagnetic center. Electrochemical studies show that the Mo_2_^4+^/Mo_2_^5+^ redox couple responds to the electronic characteristics of the axial benzoate para substituent (σ_p_), with the *E*_1/2_ for the Mo_2_^4+^/Mo_2_^5+^ redox couple increasing with substituent σ_p_. We also demonstrate that these synthetic methods are viable for ligands beyond the para-substituted benzoate series through the preparation and characterization of a Mo_2_Ni­(dpa)_4_(O_2_CFc)_2_ complex, **2**, where O_2_CFc = ferrocene carboxylate, a redox-active ligand.

## Graphical abstract

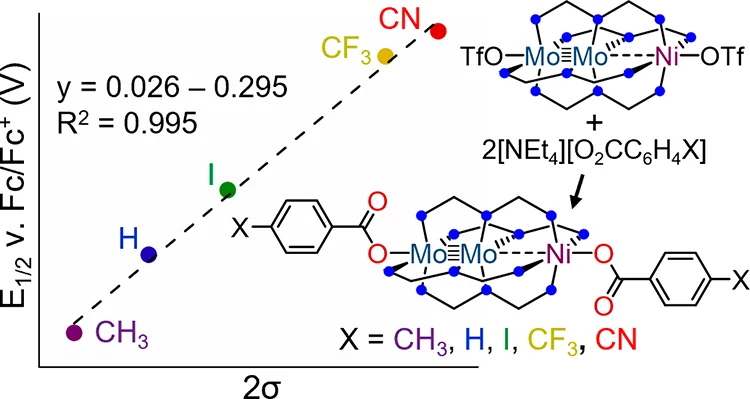

## Introduction

Multimetallic systems are of current interest to the inorganic and materials community for their magnetic and spectroscopic qualities,^1–3^ as well as their potential applications as molecular-scale electronic components.^4–8^ Our group has studied a unique class of multimetallic compounds known as heterometallic extended metal atom chains (HEMACs) that feature linear, metal–metal bonded arrays of metal atoms.^2,9^ The HEMAC complexes in this work have a chemical formula of M≣M–M′(dpa)_4_X_2_, where M≣M represents a metal–metal quadruple-bonded pair of group VI metals, M′ is a heterometal ion, in most cases a paramagnetic first-row transition metal ion, X is a capping anionic axial ligand, and dpa is 2,2′-dipyridylamide.

HEMAC complexes with axial halide ligands are by far the most synthetically accessible, since they can be prepared by the addition of M′X_2_ halides to M_2_(dpa)_4_ metalloligands. A similar strategy using M′(OTf)_2_ precursors was used to prepare the Mo_2_M′(dpa)_4_(OTf)_2_ series,^10^ while the preparation of HEMACs with X = N_3_^11^, NCS, or NCSe axial ligands^12^ required the displacement of labile ligands such as triflate or acetonitrile.^11,13^ Notably, we had good success in the preparation of Mo_2_Ni­(dpa)_4_X_2_ type compounds with axial pseudohalides by using Mo_2_Ni­(dpa)_4_(OTf)_2_ as a HEMAC precursor (Figure 1, top), for two reasons. The first had to do with the experimental parameters used for the substitution reaction, which utilized strictly stoichiometric amounts of incoming X^–^ and low temperatures (0 °C) to prevent the demetalation of M′ from the HEMAC metal-atom chain and metal atom scrambling within the chain. Second, the Mo–Mo–Ni metal atom chain is the most covalent of the heterometallic chains we have studied^10^ which further works to prevent demetalation and scrambling.

**Figure 1:**
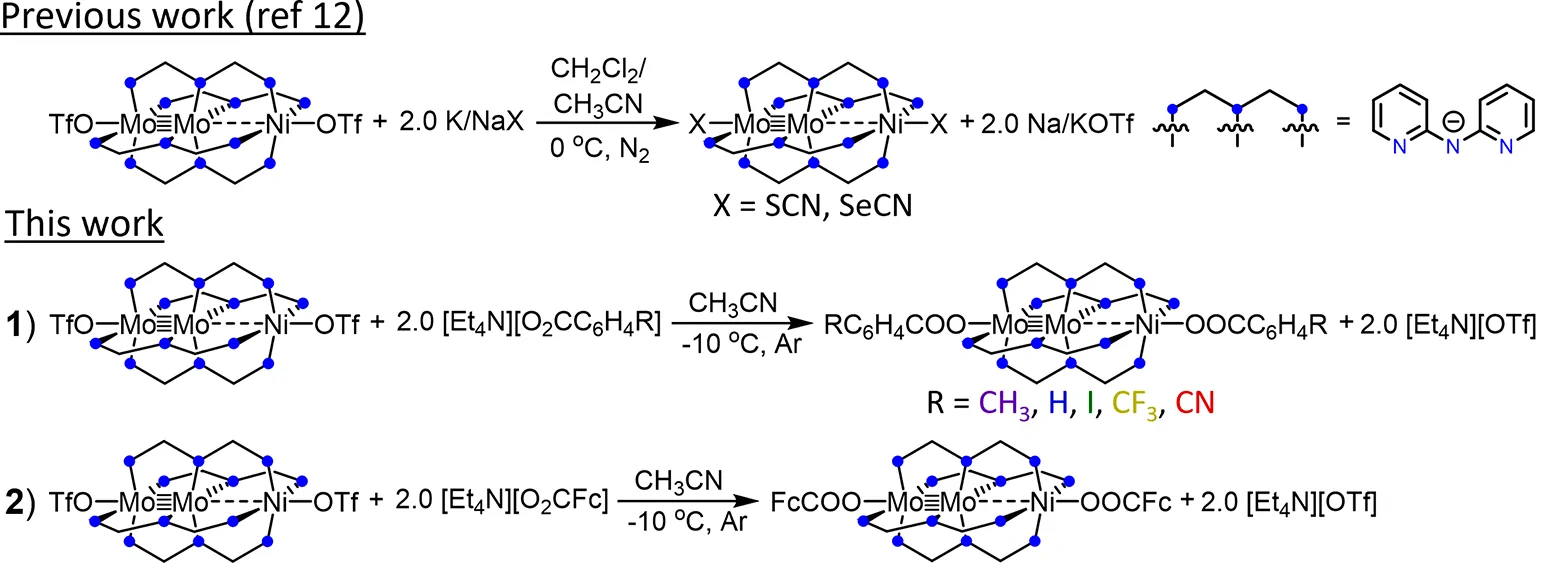
An overview of the HEMAC complexes discussed in this work and the optimized synthetic routes used to prepare them.

We therefore hypothesized that these synthetic methods could be extended to other anionic ligands and provide a synthetic entry point for the synthesis of novel multimetallic complexes or even coordination-polymer materials. As an operational test of this hypothesis, we chose to explore the synthesis of HEMACs with carboxylate (O_2_CX) axial ligands as synthetic targets for several reasons. First, carboxylates are widely employed within the realms of both synthetic organic and inorganic chemistry because they provide a systematic way to probe the electronic sensitivity of molecular systems.^14–16^ The development of synthetic methods to install axial ligands with specific electron-donating vs -withdrawing characteristics would provide an additional handle for us to explore the nature of the metal–metal bonding present in HEMACs. Second, a wide range of carboxylates are either commercially available or straightforward to prepare, and thus access to carboxylate axial ligands would dramatically expand the scope of synthetically accessible HEMAC complexes.

Here, we report the preparation and properties of a new systematic series of Mo_2_Ni­(dpa)_4_(O_2_CC_6_H_4_X)_2_ type HEMAC complexes, as shown in Figure 1 having substituted benzoate axial ligands where X is a para substituent: CH_3_ (**1_CH**_**3**_), H (**1_H**), I (**1_I**), CF_3_ (**1_CF**_**3**_), or CN (**1_CN**). We found that, despite our previous success with axial ligand substitutions involving pseudohalide Lewis bases, the synthesis of series **1** was not straightforward and required a further, extensive exploration of the synthetic space surrounding HEMAC axial ligand substitution. A combination of crystallographic characterization, electronic absorption spectroscopy, and SQUID magnetometry indicates that the complexes in series **1** possess similar structures in both the solid state and solution and contain high spin, *S* = 1 Ni^2+^ paramagnetic centers. However, despite the structural and electronic similarities, electrochemical studies of series **1** demonstrate a cathodic shift in the Mo_2_^4+^/Mo_2_^5+^ redox couple that corresponds to the electronic characteristics of the axial benzoate para substituent, σ_p_.^15^ We also demonstrate that this synthesis is extendable beyond that of substituted benzoates through the preparation of Mo_2_Ni­(dpa)_4_(O_2_CFc)_2_ (**2**), where Fc = ferrocene, which is similarly characterized in this work.

## Experimental Methods

### Materials and General Methods

All reactions were carried out under a dry N_2_ atmosphere using Schlenk techniques and glovebox methods. Anhydrous hexanes, toluene, and diethyl ether were purchased from Sigma-Aldrich. Acetonitrile was freshly distilled from CaH_2_ under an N_2_ atmosphere. MeOH was freshly distilled from Mg/I_2_ under an N_2_ atmosphere. All solvents were stored over 3 Å molecular sieves under an N_2_ atmosphere for at least 3 days before use. Tetraethylammonium hydroxide was utilized in the form of a 35 wt % solution in methanol, and all the carboxylic acid precursors were purchased from Sigma-Aldrich and used as received. Eicosane was purchased from Sigma-Aldrich, melted and fused four times under vacuum, and stored under an atmosphere of N_2_. Bu_4_NBF_4_ was purchased from Sigma-Aldrich and dried under vacuum at 50 °C for 24 h prior to use. Mo_2_Ni­(dpa)_4_(OTf)_2_ was prepared as previously described in the literature. IR spectra were taken on a Bruker Tensor 27 FTIR spectrometer using an attenuated total reflectance (ATR) adapter under an atmosphere of Ar. Elemental analyses were carried out by Midwest Microlabs, LLC, in Indianapolis, IN. MALDI mass spectra were obtained on a Bruker Impact II MALDI mass spectrometer. ^1^H and ^13^C NMR spectra of the [NEt_4_]­[O_2_CC_6_H_4_X] and [NEt_4_]­[OOCFc] salts were obtained on a 500 MHz Bruker Advance III spectrometer at 298 K in CD_3_CN solvent. ^1^H NMR spectra of the series **1** were obtained on a 500 MHz Bruker NEO spectrometer at 298 K in C_6_D_6_ solvent.

## Physical Measurements

For variable-temperature magnetic susceptibility measurements, polycrystalline samples of **1_CH**_**3**_, **1**_**H**, and **1**_**CF**_**3**_ were suspended in eicosane in polycarbonate sample holders and mounted in a SQUID magnetometer (Quantum Design MPMS 3). DC data were measured in the temperature range of 1.8–300 K with an applied field of 0.1 T. Variable-temperature/variable-field measurements of the same samples of **1_CH**_**3**_, **1**_**H**, and **1**_**CF**_**3**_ were also collected at 2, 4, 6, and 8 K, with the field swept from 0.1 to 7 T. Experimental susceptibility data were corrected for the underlying diamagnetism using Pascal’s constants.^17^ All data were modeled using the program PHI,^18^ and parametrized using the following general formalism of the spin Hamiltonian:

$$Ĥ=\beta \overset{⇀}{S}g\overset{⇀}{H}+\overset{⇀}{S}D\overset{⇀}{S}$$

where β is the Bohr magneton, $\overset{⇀}{H}$ is the applied external magnetic field, and $\overset{⇀}{S}$ , ***g***, and ***D*** are the spin vector, electronic **g**-tensor, and zero-field splitting (ZFS) tensors, respectively. The ***D*** tensor was parametrized with an axial component, $D=\frac{3}{2}D_{zz}$ , and a rhombic component, $E=\frac{1}{2}\left(D_{xx}-D_{yy}\right)$ . Fits including rhombicity provided values of *E* that were chemically unreasonable, and therefore, *E* was excluded from models of the magnetic data **1_CH**_**3**_, **1**_**H**, and **1**_**CF**_**3**_. Simultaneous fits of the susceptibility and reduced magnetization data allowed for the assignment of the sign of *D* > 0 for all three compounds. A temperature-independent paramagnetism (TIP) contribution to the experimental susceptibility was included as a parameter in the susceptibility models for all three compounds.

## Electrochemical Methods

### Preparation of [Bu_4_N]­[BF_4_]·3PhCH_3_

The preparation of [Bu_4_N]­[BF_4_]·3PhCH_3_ is described elsewhere.^19–21^ Briefly, PhCH_3_ (30 mL) and Bu_4_NBF_4_ (15 g) were combined in a Schlenk flask and allowed to stir overnight at 30 °C, resulting in the phase separation of ∼15 mL of toluene and ∼20 mL of the immiscible bottom layer corresponding to [Bu_4_N]­[BF_4_]·3PhCH_3_.

### Electrochemical Studies

The cyclic voltammetry samples were prepared using approximately 5–8 mg of **1**_**CH**_**3**_, **1_H**, **1_I**, **1_CF**_**3**_, **1_CN**, or **2** in approximately 4–5 mL of [Bu_4_N]­[BF_4_]·3PhCH_3_ with 1–2 mL of toluene layered on top of the [Bu_4_N]­[BF_4_]·3PhCH_3_/analyte solution. While the additional toluene layer is required to prevent precipitation of the electrolyte, it prevented us from accurately determining the concentration of the complexes in each sample. Note that [Bu_4_N]­[BF_4_] can precipitate from solution at 22 °C which can make obtaining a cyclic voltammogram difficult.^20^ We warmed our samples to 25 °C to prevent the precipitation of [Bu_4_N]­[BF_4_].

The electrochemical measurements were performed under a N_2_ atmosphere in a glovebox. Cyclic voltammograms were collected by using a glassy carbon disk electrode and a reference electrode consisting of a silver wire and a 10 mM AgNO_3_ solution in MeCN, with a 100 mV/s scan rate. The internal reference of ferrocene was used to determine the *E*_1/2_ values of each complex measured.

### Synthetic Methods

Safety statement: no uncommon hazards were noted with the preparation of the [NEt_4_]­[O_2_CC_6_H_4_X]-type salts, [NEt_4_]­[O_2_CFc], series **1**, and compound **2**.

All [NEt_4_]­[O_2_CC_6_H_4_X] salts and the [NEt_4_]­[O_2_CFc] salt were prepared using similar general procedures, which are described only for [NEt_4_]­[O_2_CC_6_H_5_].

**[NEt_4_]­[O_2_CC_6_H_5_]**. White HO_2_CC_6_H_5_ (0.729 g, 5.97 mmol) was dissolved in 5 mL of MeOH. A 4.4 mL aliquot of a 35% wt solution of [NEt_4_]­[OH] in MeOH was then added to the colorless solution, and the mixture was allowed to stir for 30 min under an atmosphere of N_2_, followed by an additional 30 min of stirring in air. The colorless solution was then reduced to dryness under vacuum, affording a white reside. The white residue was extracted into 30 mL of CH_3_CN and filtered over Celite, producing a clear, colorless solution. Reducing this second solution to dryness under vacuum affords the [NEt_4_]­[O_2_CC_6_H_5_] salt as a white hygroscopic powder. These initial steps were followed by recrystallizing the crude product in a 20:1 PhCH_3_:CH_3_CN solution at 70 °C, followed by drying the resulting white powder at 40 °C for 24 h to afford analytically pure samples of the [NEt_4_]­[O_2_CC_6_H_5_]. Yield: 0.692 g, 46.0%. ^1^H NMR (500 MHz, CD_3_CN) δ (ppm): 1.21 *t* (12H, *J* = 10 Hz), 3.18 *q* (8H, *J* = 10 Hz), 7.25 *m* (3H), 7.91 *m* (2H). ^13^C NMR (125 MHz, CD_3_CN) δ (ppm): 7.28, 52.67 *t* (*J*(^15^N) = 3.1 Hz), 127.48, 128.41, 129.63, 143.08, 169.66. IR (ATR, cm^–1^) 3460 w, 3301 w, 3004 w, 1735 w, 1608 s, 1596 s, 1560 s, 1487 m, 1397 m, 1367 s, 1352 m, 1178 m, 1007 m, 825 w, 789 m, 738 s, 669 w.

[**NEt_4_]­[O_2_CC_6_H_4_CH_3_]**. **Yield:** 0.744 g white powder, 64.1%. ^1^H NMR (500 MHz, CD_3_CN) δ (ppm): 1.24 *t* (12H *J* = 10 Hz), 2.36 *s* (3H, CH_3_), 3.23 *q* (8H, *J* = 10 Hz), 7.12 *m* (2H), 7.85 *m* (2H). ^13^C NMR (125 MHz, CD_3_CN) δ (ppm): 7.31, 20.88, 52.66 *t* (*J*(^15^N) = 3.1 Hz), 128.20, 129.76, 138.28, 139.86, 170.07. IR (ATR, cm^–1^) 1654 w, 1611 s, 1567 m, 1487 w, 1396 w, 1358 w, 1324 s, 1162 s, 1114 s, 1100 s, 1062 s, 1015 w, 999 w, 872 w, 838 w, 794 s, 713 s, 679 w.

**[NEt_4_]­[O_2_CC_6_H_4_I]**. **Yield:** 0.489 g white powder, 49.5%. ^1^H NMR (500 MHz, CD_3_CN) δ (ppm): 1.12 *t* (12H, *J* = 10 Hz), 3.08 *q* (8H, *J* = 10 Hz), 7.52 *m* (2H), 7.60 *m* (2H). ^13^C NMR (125 MHz, CD_3_CN) δ (ppm): 7.26, 52.67 *t* (*J*(^15^N) = 3.1 Hz), 94.24, 131.98, 136.57, 142.94, 168.51. IR (ATR, cm^–1^) 1594 s, 1580 m, 1549 s, 1487 m, 1392 m, 1357 s, 1174 w, 1010 m, 832 w, 820 s, 780 s, 694 w, 649 w, 639 w.

**[NEt_4_]­[O_2_CC_6_H_4_CF_3_]**. **Yield:** 0.922 g white powder, 61.0%. ^1^H NMR (500 MHz, CD_3_CN) δ (ppm): 1.23 *t* (12H, *J* = 10 Hz), 3.22 *q* (8H, *J* = 10 Hz), 7.59 *m* (2H), 8.09 *m* (2H). ^13^C NMR (125 MHz, CD_3_CN) δ (ppm): 7.29, 52.67 *t* (*J*(^15^N) = 3.1 Hz), 124.47 (q, *J*(^19^F) = 3.8 Hz), 125.94 (q, *J*(^19^F) = 270.5 Hz), 129.48 (q, *J*(^19^F) = 31.4 Hz), 130.00, 147.07, 168.07. IR (ATR, cm^–1^) 1729 w, 1598 s, 1556 m, 1489 m, 1454 m, 1441 m, 1398 m, 1359 s, 1175 m, 1056 w, 1006 m, 826 w, 774 s, 754 m, 703 m, 654 w, 638 m, 603.

**[NEt_4_]­[O_2_CC_6_H_4_CN]**. **Yield:** 0.602 g white powder, 39.2%. ^1^H NMR (500 MHz, CD_3_CN) δ (ppm): 1.20 *t* (12H, *J* = 10 Hz), 3.20 q (8H, *J* = 10 Hz), 7.61 *m* (2H), 8.02 *m* (2H). ^13^C NMR (125 MHz, CD_3_CN) δ (ppm): 7.31, 52.66 *t* (*J*(^15^N) = 3.1 Hz), 111.43, 120.01, 130.12, 131.76, 147.63, 167.75. IR (ATR, cm^–1^) 3239 w, 1684 w, 1595 s, 1555 m, 1485 m, 1412 w, 1396 m, 1364 s, 1174 w, 807 w, 783 w, 770 m, 729 m.

**[NEt_4_]­[O_2_CFc]**. **Yield:** 0.401 g orange powder, 38.3%. ^1^H NMR (500 MHz, CD_3_CN) δ (ppm): 1.21 *s*, 3.20 *s*, 4.02 *s*, 4.04 *s*, 4.43 *s*. ^13^C NMR (125 MHz, CD_3_CN) δ (ppm): 7.37, 52.72, 68.27, 69.04, 70.37, 86.02, 171.78.

Note: The aliphatic ^1^H resonance integrations corresponding to the NEt_4_^+^ protons for the [NEt_4_]­[O_2_CC_6_H_4_CN] and the [NEt_4_]­[O_2_CC_6_H_4_I] salts are substantially above the expected values of 8 and 12. While all salts were extracted and recrystallized, this discrepancy is likely due to a small [NEt_4_]_2_[CO_3_] impurity that is the source of the larger-than-expected integrations. We were unable to separate the [NEt_4_]_2_[CO_3_] impurity from the product compounds, and attempts at using substoichiometric [NEt_4_]­[OH] resulted in a remainder of unreacted and inseparable carboxylic acid starting material. We elected to use the [NEt_4_]­[O_2_CC_6_H_4_X] type salts as reported here for preparation of the trimetallic complexes, which we note show no sign of a CO_3_^2–^ impurity by NMR, EA, or other spectroscopies.

Compounds **1_CH_3_**, **1_H**, **1_I**, **1_CF**_**3**_, **1_CN**, and **2** were prepared using analogous synthetic procedures; details are provided only for **1_H**.

**Mo_2_Ni­(dpa)_4_(O_2_CC_6_H_5_)_2_ (1_H)**. Dark green crystals of Mo_2_Ni­(dpa)_4_(OTf)_2_ (0.133 g, 0.108 mmol) and white [NEt_4_]­[O_2_CC_6_H_5_] (0.0543 g, 0.216 mmol) were separately dissolved in 15 and 5 mL of CH_3_CN affording dark green and colorless solutions, respectively. Both solutions were allowed to sit at −10 °C for ca. 30 min, after which time the [NEt_4_]­[O_2_CC_6_H_5_] solution was added to the Mo_2_Ni­(dpa)_4_(OTf)_2_ dropwise. Progressive color changes from green to red to finally dark yellow-brown, with a dark brown precipitate, were observed over the course of the addition. The dark gold suspension was stirred for an additional 10 min and then reduced to dryness under vacuum, forming a gold-brown residue, which was then allowed to dry for an additional hour under vacuum at room temperature. The dark gold residue was then extracted into 12 mL of cold PhCH_3_ (at −78 °C) and filtered over a medium porosity frit, affording a dark gold solution. This solution was layered with 100 mL of cold hexane (also at −78 °C) and left for 7 days at −78 °C while crystals of the product grew. Yield: 46 mg, 36% dark green/yellow crystals. ^1^H NMR (500 MHz, C_6_D_6_): δ (ppm) 71.41 (s), 31.39 (s), 17.34 (s), 16.09 (s), 15.24 (s), 13.14 (s), 12.83 (s), 9.92 (s), 9.40 (s), 8.94 (s), 8.52 (s), 8.00 (s), 6.31 (s), 3.57 (s). IR (ATR, cm^–1^) 1603 m, 1593 m, 1572 w, 1461 s, 1421 s, 1341 s, 1311 w, 1263 s, 1224 w, 1155 m, 1031 w, 1017 w, 857 w, 768 s, 732 m, 718 s, 695 w, 669 w, 637 m. MALDI-MS *m*/*z* 1052.45 [M–O_2_CC_6_H_5_]^+^. Anal. Calcd for Mo_2_Ni­(dpa)_4_(O_2_CC_6_H_5_)_2_·C_7_H_8_: C, 57.89; H 3.98; N, 13.28. Found: C, 57.71; H, 4.21; N, 11.77.

**Mo_2_Ni­(dpa)_4_(O_2_CC_6_H_4_CH_3_)_2_** (**1_CH**_**3**_): Yield: 71.9 mg, 66.0% dark gold crystals. ^1^H NMR (500 MHz, C_6_D_6_): δ (ppm) 71.12 (s), 27.74 (s), 15.34 (s), 13.99 (s), 12.43 (s), 9.76 (s), 9.12 (s), 8.22 (s), 8.41 (s), 6.61 (s), 4.19 (s, b) 3.79 (s). IR (ATR, cm^–1^) 1602 s, 1593 s, 1560 w, 1464 s, 1422 s, 1340 s, 1310 m, 1281 w, 1262 w, 1155 m, 1018 m, 856 w, 765 s, 751 w, 741 m, 696 w. MALDI-MS *m*/*z* 1066 [M–O_2_CC_6_H_4_CH_3_]^+^. Anal. Calcd for Mo_2_Ni­(dpa)_4_(O_2_CC_6_H_4_CH_3_)_2_·1.5 C_7_H_8_: C, 59.61; H, 4.36; N, 12.54. Found: C, 58.79; H, 4.24; N, 11.95.

**Mo_2_Ni­(dpa)_4_(O_2_CC_6_H_4_I)_2_** (**1_I**): Yield: 35.8 mg, 29.0% dark gold crystals. ^1^H NMR (500 MHz, C_6_D_6_): δ (ppm) 71.23 (s), 27.69 (s), 15.28 (s), 13.98 (s), 12.41 (s), 9.95 (s), 8.85 (s), 8.62 (s), 8.09 (s), 6.59 (s), 6.63 (s), 3.95 (s). IR (ATR, cm^–1^) 1602 m, 1592 m, 1557 w, 1463 s, 1421 s, 1338 s, 1310 m, 1261 m, 1154 m, 1090 w, 1056 w, 1017 m, 1007 s, 855 w, 802 m, 763 s, 742 m. MALDI-MS *m*/*z* 1178 [M–O_2_CC_6_H_4_I]^+^. Anal. Calcd for Mo_2_Ni­(dpa)_4_(O_2_CC_6_H_4_I)_2_·1/2 C_7_H_8_: C, 46.94; H, 3.01; N, 11.42. Found: C, 46.91; H, 2.87; N, 11.10.

**Mo_2_Ni­(dpa)_4_(O_2_CC_6_H_4_CF_3_)_2_** (**1_CF**_**3**_): Yield: 45.9 mg, 42.0% dark green-yellow crystals. ^1^H NMR (500 MHz, C_6_D_6_): δ (ppm) 71.24 (s), 27.34 (s), 15.28 (s), 12.38 (s), 9.63 (s), 9.14 (s), 8.59 (s), 8.41 (s), 6.38 (s), 3.95 (s), 1.96 (s), 0.43 (s). ^19^F NMR (400 MHz, C_6_D_6_): δ (ppm) −171.16 (s), 193.64 (s). IR (ATR, cm^–1^) 1622 w, 1603 m, 1594 m, 1573 w, 1464 s, 1421 s, 1346 s, 1321 s, 1262 w, 1155 s, 1126 m, 1098 w, 1064 w, 1017 s, 857 w, 788 w, 766 m, 751 w, 742 m, 707 w. MALDI-MS *m*/*z* 1120 [M–O_2_CC_6_H_4_CF_3_]^+^. Anal. Calcd for Mo_2_Ni­(dpa)_4_(O_2_CC_6_H_4_CF_3_)_2_·1/2 C_7_H_8_: C, 52.72; H, 3.27; N, 12.40. Found: C, 52.17; H, 3.03; N, 12.15.

**Mo_2_Ni­(dpa)_4_(O_2_CC_6_H_4_CN)_2_** (**1_CN**): Yield: 35.7 mg, 27.0% dark gold crystals. ^1^H NMR (500 MHz, C_6_D_6_): δ (ppm) 71.20 (s), 27.87 (s), 15.34 (s), 12.45 (s), 9.63 (s), 9.42 (s), 8.97 (s), 8.66 (s), 8.34 (s), 6.34 (s), 3.94 (s), 0.36 (s). IR (ATR, cm^–1^) 2228 w, 1620 m, 1603 s, 1593 s, 1559 w, 1464 s, 1422 s, 1340 s, 1310 m, 1282 w, 1261 w, 1155 m, 1108 w, 1058 w, 1017 m, 858 w, 781 m, 766 s, 751 w, 741 m, 691 w, 640 w. MALDI-MS *m*/*z* 1077 [M–O_2_CC_6_H_4_CN]^+^. Anal. Calcd for Mo_2_Ni­(dpa)_4_(O_2_CC_6_H_4_CN_2_)·1/3 C_7_H_8_: C, 55.64; H, 3.40; N, 15.73. Found: C, 55.00; H, 3.38; N, 15.07.

**Mo_2_Ni­(dpa)_4_(O_2_CFc)_2_** (**2**): Yield: 34.0 mg, 25.0% dark gold crystals. IR (ATR, cm^–1^) 2920 w, 1600 m, 1603 s, 1589 m, 1465 s, 1421 s, 1377 w, 1350 m, 1325 s, 1261 w, 1155 m, 1108 w, 1058 w, 1016 m, 856 w, 769 s, 738 m, 698 w, 636 w. MALDI-MS *m*/*z* 1160 [M–O_2_CC_6_H_4_Fc]^+^. Anal. Calcd for Mo_2_Ni­(dpa)_4_(O_2_CC_6_H_4_Fc_2_)·1/2 C_7_H_8_: C, 54.61; H, 3.81; N, 11.76. Found: C, 54.07; H, 3.98; N, 11.18.

## Results and Discussion

### Synthesis

Initial attempts were made to install carboxylates via the reaction of Mo_2_(dpa)_4_ and Ni­(OAc)_2_ in molten naphthalene. However, the only crystals isolated from these reactions contained Mo_2_(dpa)_3_(OAc), a surprising result of equatorial ligand substitution rather than trimetallic formation (Figure S25).

We then investigated a stepwise approach based on our prior report on the substitution of Mo_2_Ni­(dpa)_4_(OTf)_2_ (**3**) with axial NCS or NCSe ligands.^12^ In this previous study, avoiding demetalation of the Ni required the slow addition of a CH_3_CN solution of the incoming ligand in strict stoichiometry as a sodium or potassium salt to a CH_2_Cl_2_ solution of **3** at low temperatures (0 °C).^12^ We encountered further restrictions when attempting to prepare HEMACs with axial carboxylate ligands. Reactions performed in CH_2_Cl_2_ yielded Mo_2_Ni­(dpa)_4_Cl_2_ (**4**) with the Cl^–^ atoms obtained presumably from the CH_2_Cl_2_ solvent. The use of Li, Na, and K carboxylate salts was explored, but these are only soluble in polar, protic solvents (MeOH and EtOH), which react with **3** to cause either demetalation (formation of Mo_2_(dpa)_4_) or oxidation (formation of [Mo_2_(dpa)_4_]­(OTf)). We switched to [NBu_4_]^+^ carboxylate salts and saw productive reactivity but found it impossible to separate the HEMAC product from the [NBu_4_]^+^[OTf]^−^ byproduct.

Ultimately, we optimized a synthetic route for the preparation of series **1** and complex **2** using [NEt_4_]­[O_2_CC_6_H_4_X] (X = variable para substituent, see Figure 1) or [NEt_4_]­[O_2_CFc] salts. These salts allow the reaction with **3** to be performed in polar but not protic solvents (e.g., CH_3_CN) and afford a [NEt_4_]­[OTf] byproduct that is insoluble in nonpolar solvents such as PhCH_3_, which can be used to extract the product **1**. The [NEt_4_]­[O_2_CC_6_H_4_X] salts are not commercially available except for the X = H salt and were prepared by deprotonation of the corresponding benzoic acid with [NEt_4_]­OH.

The optimized synthetic procedure for carboxylate axial substitution thus involves the combination of **3** and the [NEt_4_]­[O_2_CC_6_H_4_X] salt of choice in CH_3_CN at −10 °C, forming the axially substituted HEMAC as a precipitate. The resulting product is then isolated by filtration and crystallized from PhCH_3_/hexane. The final challenge we encountered was crystallizing the series **1** and complex **2**, as the compounds in solution are thermally sensitive and decompose into Mo_2_(dpa)_4_ and a monometallic Ni^2+^ product if left at room temperature. We grew X-ray quality crystals at reduced temperatures (−40 °C).

### Crystallography

Crystallographic data for **1_CH**_**3**_, **1_H**, and **1_CF**_**3**_ are presented in Table S1 and the molecular structures in Figure 2. Crystallographic intensity data of **1_I**, **1_CN**, and **2** suffered from diffuse diffraction, likely due to disordered solvent of crystallization. The data unambiguously confirm the structure connectivity, but we refrain from including metrics derived from the poor structural models in the discussion below.

**Figure 2:**
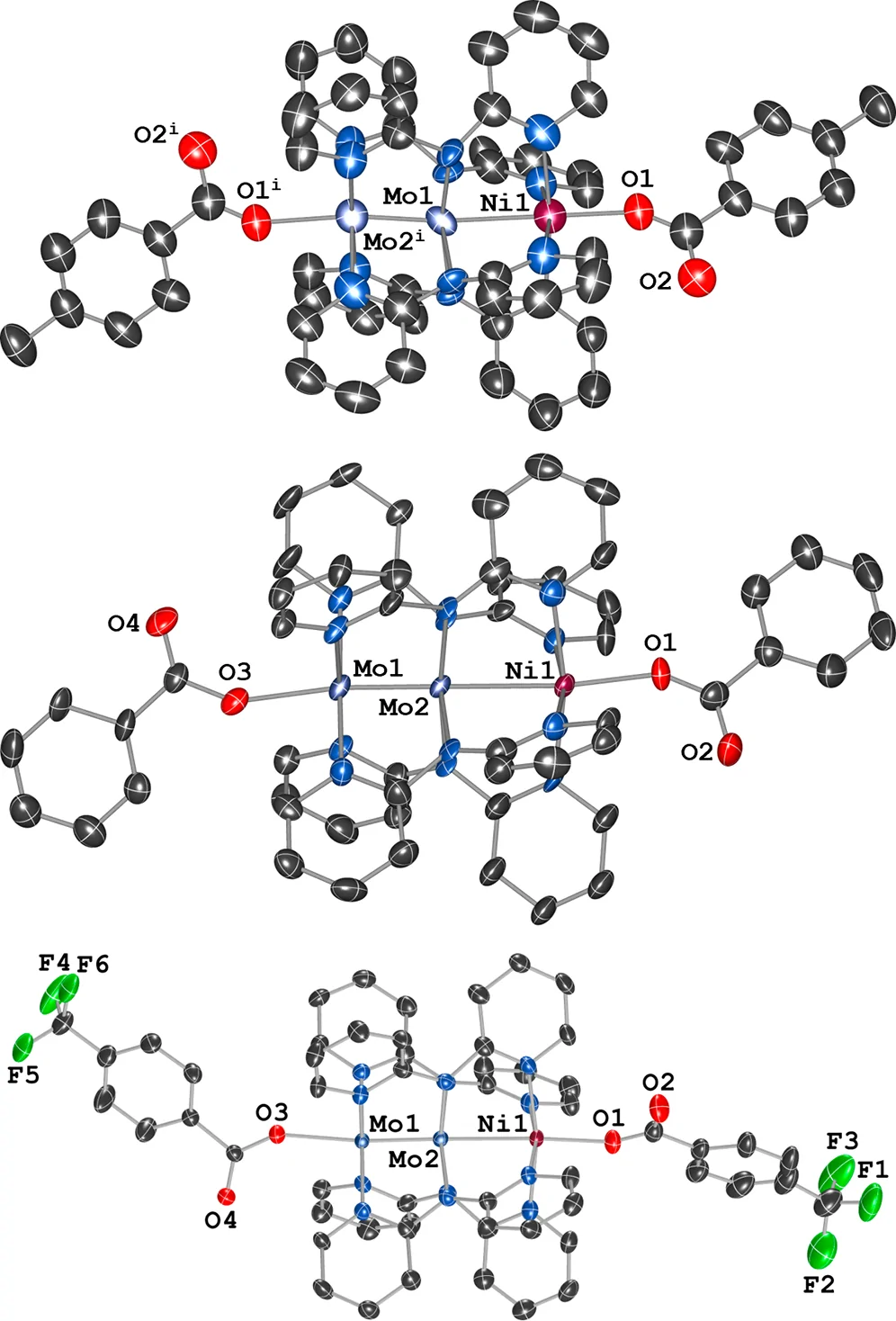
Molecular drawings of **1_CH_3_** (top, [Symmetry code: (i) 1–x, 1–y, z.], **1**_**H** (middle), and **1_CF_3_** (bottom) shown with 50% probability ellipsoids and selected atom labels. All H atoms, solvent molecules, and minor disorder components are omitted for clarity. Additional molecular drawings are provided in the Supporting Information.

The complexes **1_CH**_**3**_, **1_H**, and **1_CF**_**3**_ all crystallize with similar molecular formulas, with two to three solvent toluene molecules. However, they all crystallize in different crystal systems and space groups – *P*4̅2_1_ *a* for **1_CH**_**3**_, *Pna*2_1_ for **1_H**, and *P*2_1_/*c* for **1_CF**_**3**_ – despite the formulaic similarities. Additionally, all three complexes present disorder of the central amide N atoms of the dpa ligand and the metal-atom chain, whose crystallographic models are discussed in the Supporting Information.

The geometric parameters for **1_CH**_**3**_, **1_H**, and **1_CF**_**3**_ are presented in Table 1, as well as the geometric parameters for **3** and **4**·2 CH_2_Cl_2_. All three structures contain relatively similar geometric characteristics. The Mo–Mo distances range from 2.106(3) to 2.110(9) Å, consistent with the presence of a Mo≣Mo quadruple bond. The Mo_2_–Ni distances of **1_CH**_**3**_, **1_H**, and **1_CF**_**3**_ are all less than the sum of the Mo and Ni van der Waals radii (4.85 Å).^23^ These distances are similar to those of the previously reported Mo_2_Ni­(dpa)_4_X_2_ (X = NCS, NCSe) HEMACs as well as compounds **3** and **4**. In those cases, electronic structure analysis facilitated by density functional theory (DFT) calculations indicated that there is a 3c-3e^–^ σ bond between the three metal atoms in the HEMAC complex,^2,10,12^ and the structures (as well as other electronic features, vide infra) of series **1** are consistent with this assignment as well. The Mo–O and Ni–O distances of **1_CH**_**3**_, **1_H**, and **1_CF**_**3**_ are shorter (with the exception of the Ni–L_ax_ distance for **1_CH**_**3**_) than those of **3** due to the superior donating characteristics of the benzoate vs OTf ligand.

**Table 1:** Selected Crystallographic Bond Distances for 1_CH_3_, 1_H, 1_CF_3_, 3, and 4^a^ ^,^ ^c^

	^b^ **1_CH_3_** **·**PhCH_3_	^b^ **1_H** **·**3PhCH_3_	^b^ **1_CF_3_** **·**2.87 PhCH_3_	**3**	**4** **·**2 CH_2_Cl_2_
Mo–Mo (Å)	2.101(10)	2.106(5)	2.101(3)	2.110(8)	2.107(2)
Mo–Ni (Å)	2.549(16)	2.582(9)	2.571(9)	2.485(8)	2.525(4)
Ni–N (Å)	2.151[3]	2.156[5]	2.133[2]	2.157[1]	2.146[3]
Mo_i_–N (Å)	2.124[6]	2.120[4]	2.1260[11]	2.114[2]	2.115[3]
Mo_o_–N (Å)	2.144[5]	2.165[3]	2.171[7]	2.157[5]	2.174[3]
Mo–L_ax_ (Å)	2.249(8)	2.223(8)	2.279(4)	2.364(4)	2.627(2)
Ni–L_ax_ (Å)	2.083(14)	2.044(9)	2.042(9)	2.086(5)	2.394(3)
ref.	This work	This work	This work	^22^	This work

a Mo_o_/Mo_i_ = outer/inner Mo atom; L_ax_ = O_2_CC_6_H_4_X for **1_CH**_**3**_, **1_H**, and **1_CF**_**3**_, OTf^–^ for **3**, and Cl^–^ for **4**.

b In instances of metal atom chain disorder, the majority position was used for geometric parameters.

c The (*x*) notation is used to denote standard uncertainties obtained from a single symmetry-independent distance, and the [*x*] notation is used for combined standard uncertainties (i.e., the root-mean-square of the uncertainties from multiple symmetry-independent distances scaled by the structure goodness of fit).

Comparing within the series **1_CH**_**3**_, **1_H**, and **1_CF**_**3**_, the electron-donating characteristics of the *para*-benzoate axial ligand decrease from **1_CH**_**3**_ > **1_H** > **1_CF**_**3**_. Rather than reflecting this electronic trend, the metal–oxygen distances more likely reflect crystal packing effects (vide infra). For instance, the Mo–O distances are longer than the Ni–O distances on average by only 0.19 Å, which is smaller than the difference of the single bond covalent radii for Mo (1.38 Å) and Ni (1.10 Å).^24^ Thus, we do not observe a trans-influence from the Mo–Mo multiple bond as observed with other HEMAC structures.^25^ Additionally, all three structures crystallize with one to three molecules of toluene. Inspection of intermolecular packing for **1**_**CH**_**3**_ (before application of Platon SQUEEZE),^100^ **1**_**H**, and **1**_**CF**_**3**_ show the presence of several types of nonclassical hydrogen bonding interactions between H atoms of the solvate toluene and the O (and for **1_CF**_**3**_ the CF_3_ F atoms) of the O_2_CC_6_H_4_R ligands. Additionally, **1**_**H** displays C–H···π type interactions between the H atom of tolyl aryl group and the centroid of the benzoate aryl group. These interactions (∼2.6–2.9 Å) are on the order of strength found for that of benzene-CHCl_3_.^26^ This inspection also provides a rationalization for the qualitative experimental finding that crystallization of series **1** from other solvents such as THF only produces low-quality microcrystalline material.

### Electronic Absorption Spectra

The electronic absorption spectra for series **1** and complex **2** are shown in Figure 3. All spectra, regardless of the axial ligand, are almost identical, with three major features (A, B, and C) whose λ_max_ values are provided in Table 2. The electronic absorption spectra for Mo_2_ type HEMACs are well studied,^2,10,12^ and we can assign the A and B features in these spectra as metal–ligand charge transfer from the Mo_2_ δ orbital to the dpa π* orbital. In contrast, feature C corresponds to the δ to δ* transition of the Mo≣Mo quadruple bond.

**Table 2:** Electronic Absorption Spectra with λ_max_ and Molar Absorptivities for Series **1** and **2**

	A (Mo_2_ δ → dpa π*), nm (L mol^–1^ cm^–1^)	B (Mo_2_ δ → dpa π*), nm (L mol^–1^ cm^–1^)	C (Mo_2_ δ → δ*), nm (L mol^–1^ cm^–1^)
**1_H**	415 (19,000)	493 (14,000)	664 (2,300)
**1_CF**_**3**_	414 (19,000)	492 (12,000)	659 (2,100)
**1_CH**_**3**_	412 (21,000)	493 (14,000)	659 (2,400)
**1_I**	414 (17,000)	494 (12,000)	663 (2,100)
**1_CN**	414 (22,000)	493 (14,000)	660 (2,600)
**2**	414 (22,000)	493 (14,000)	660 (2,600)

**Figure 3:**
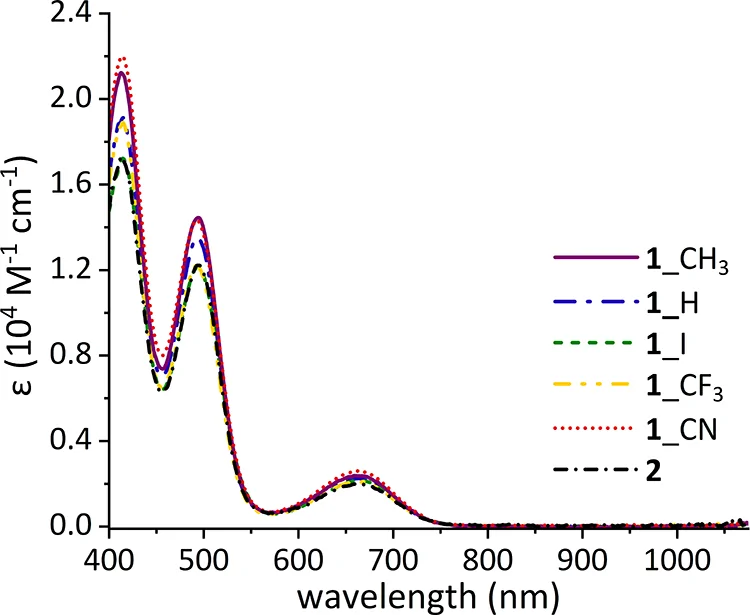
Electronic absorption spectra of 1_CH3, 1_H, 1_I, 1_CF3, 1_CN, and 2.

Previous studies reported that the absorption spectra of HEMAC complexes are relatively insensitive to identities of the heterometal atom in the trimetallic.^10^ Instead, the absorption spectra and visible colors of HEMAC complexes are largely determined by subtle shifts in the charge transfer features (A and B) that are most sensitive to structural changes around the Mo≣Mo quadruple bond, which the identity of the axial ligand only indirectly influences.^12^ The similarity of the absorption spectra (and their qualitatively identical colors in solution) for the series **1** suggests that the environment surrounding the Mo≣Mo quadruple bond is similar and relatively invariant in solution, consistent with the similarity of the crystal structures of **1_CH**_**3**_, **1**_**H**, and **1_CF**_**3**_. Thus, we may conclude that charge transfer transitions involving the axial benzoate ligands are not observed.

### SQUID Magnetometry

Variable-temperature magnetic susceptibility and variable-field magnetization isotherms were measured for **1_CH**_**3**_, **1**_**H**, and **1_CF**_**3**_; these data for **1_H** are shown in Figure 4 and those for **1_CH**_**3**_ and **1_CF**_**3**_ are shown in Figure S27. The χT values at 25 K for **1_CH**_**3**_, **1**_**H**, and **1_CF**_**3**_ are 0.99, 1.01, and 1.43 cm^3^ K mol^–1^ respectively, consistent with the presence of an *S* = 1 paramagnetic Ni­(II) center. The downtick in the susceptibility data below ∼20 K and the nonsuperimposable isotherms indicate the presence of modest zero-field splitting, which was fitted to an axial model with *D* > 0. The values of *D* were unambiguously determined to be positive, and the fitting parameters are provided in Table 3. The *g* values for the three HEMACs are near *g* = 2, though some values are less than 2, which is unusual for Ni­(II). We previously observed values of *g* < 2 before with the Mo_2_Ni­(dpa)_4_X_2_ systems (X = OTf, NCS, NCSe),^12^ which we attribute to substantial covalency of the Mo_2_–Ni chain. Here, the magnetic orbital of σ symmetry receives contributions from all three metal atoms in the HEMAC. The contributions of the two Mo atoms therefore result in the depressed values of *g*, especially the *g*_∥_ components. This is corroborated by the finding that spin density localized on the outer Mo atom in HEMACs increases with increasing Mo_2_–M covalency and maximizes for Mo_2_–Ni trimetallic chains.^10^.

**Table 3:** SQUID Fit Parameters for **1_CH_3_**, **1**_**H**, and **1_CF_3_**

	*g*_||_	***g***_⊥_	*D* (cm^–1^)	TIP (cm^–3^ mol^–1^)	Residual^a^
**1_CH**_**3**_	1.939(2)	1.92222(7)	7.619(8)	0.002667(2)	0.0000089
**1_H**	1.81(1)	2.023(3)	9.99(3)	0.002542(9)	0.000034
**1**_**CF**_**3**_	1.763(8)	2.181(3)	8.02(2)	0.01510(2)	0.000436

a Residual $=\left[\overset{points}{\underset{i=1}{\sum }}{\left(M_{\exp}-M_{calc}\right)}^{2}\right]\left[\overset{points}{\underset{i=1}{\sum }}{\left(\chi_{\exp}-\chi_{calc}\right)}^{2}\right]$ where *M*_exp_/*M*_calc_ = measured/calculated magnetization and χ_exp_/χ_calc_ = measured/calculated susceptibility

**Figure 4:**
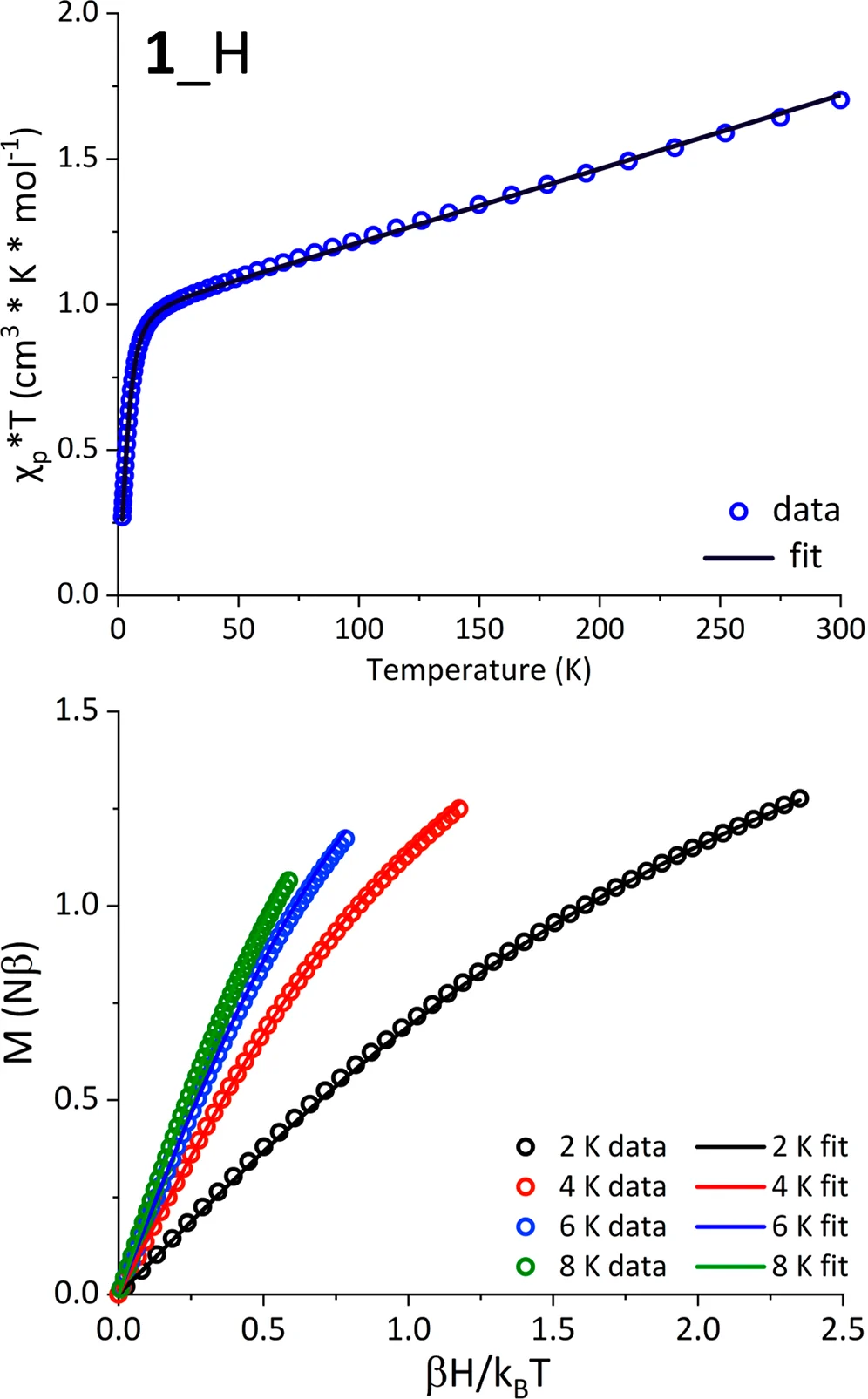
Magnetic susceptibility (top) and reduced magnetization (bottom) data and fits for **1_H**. The data and fits for **1_CH_3_** and **1_CF_3_** are provided in the Supporting Information.

**Table 4:** Electrochemical Data for Series **1** and **2** Collected at 100 mV/s in Bu_4_NBF_4_·3PhCH_3_

Compound	Ligand ∑σ	*E*_1/2_ (V)	ΔE_p_ (mV)	*I*_pc_/*I*_pc_
**1**_**CH**_**3**_	–0.34	–0.30516	67.1	0.953
**1**_**H**	0.00	–0.29411	63.8	0.887
**1**_**I**	0.36	–0.28495	92.6	0.995
**1**_**CF**_**3**_	1.08	–0.26605	79.8	1.230
**1**_**CN**	1.32	–0.26249	80.8	0.953

	Mo_2_^4+^/Mo_2_^5+^			Fc/Fc^+^		
	*E*_1/2_ (V)	ΔE_p_ (mV)	*I*_pc_/*I*_pc_	*E*_1/2_ (V)	ΔE_p_ (mV)	*I*_pc_/*I*_pc_
**2**	–0.324	65	0.816	0.138	113	n/a

### Electrochemical Studies

Complexes of series **1** are not soluble and/or are not stable in traditional solvents used in cyclic voltammetry studies; however, they are soluble and stable in PhCH_3_. We found that Bu_4_NBF_4_·3PhCH_3_ is an appropriate electrochemical medium, and its preparation is discussed in the Supporting Information.^19–21^

The cyclic voltammograms of series **1** collected in Bu_4_NBF_4_·3PhCH_3_ all show a reversible redox event with an *E*_1/2_ between −0.31 and −0.26 V vs Fc/Fc^+^ which we assign to the Mo_2_^4+^/Mo_2_^5+^ couple of the Mo≣Mo quadruple bond (Figure 5). Notably, the Mo_2_^4+^/Mo_2_^5+^ couples of series **1** are only slightly higher than that previously reported for **4** (−0.42 mV) which was successfully oxidized chemically to the metastable [Mo_2_Ni­(dpa)_4_Cl_2_]^+^ cation.^27^ The cyclic voltammogram of **2** in Bu_4_NBF_4_·3PhCH_3_ (Figure 6) is similar to those of series **1.** However, the reversible Mo_2_^4+/5+^ redox couple is more readily oxidized with *E*_1/2_ = −0.320 V in agreement with the ^–^O_2_CFc ligand being a stronger electron donor than benzoates.^28^ Compound **2** also displays a second feature from the Fc/Fc^+^ couple of the axial ^–^O_2_CFc ligands, which is broad and has approximately twice the current passed as that of the Mo_2_^4+^/Mo_2_^5+^ couple (Figures S28 and S29, Table S4). This corresponds well to the structure of **2**, where we expect two redox couples of similar but not quite identical *E*_1/2_’s from the ^–^O_2_CFc moiety binding to the outer Mo atom or the Ni heterometal of the HEMAC complex. Importantly, the fact that the Fc waves are overlapping in the cyclic voltammogram indicates that there is little communication across the metal atom chain between the ^–^O_2_CFc ligands, an assessment supported by the absence of metal-to-benzoate charge transfer features in the absorption spectra for series **1**.

**Figure 5:**
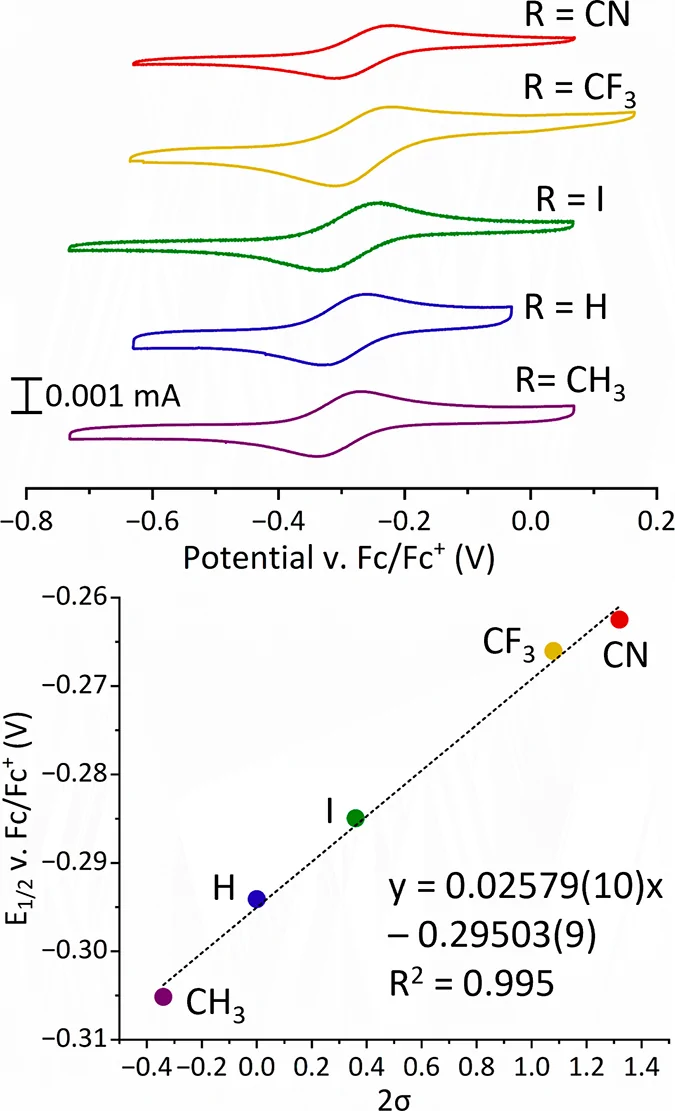
Top: cyclic voltammograms of series **1** in Bu_4_NBF_4_·3PhCH_3_. Bottom: Linear free energy relationships for the redox reaction of Mo_2_^4+^/Mo_2_^5+^ based on the series **1** reported in this work.

**Figure 6:**
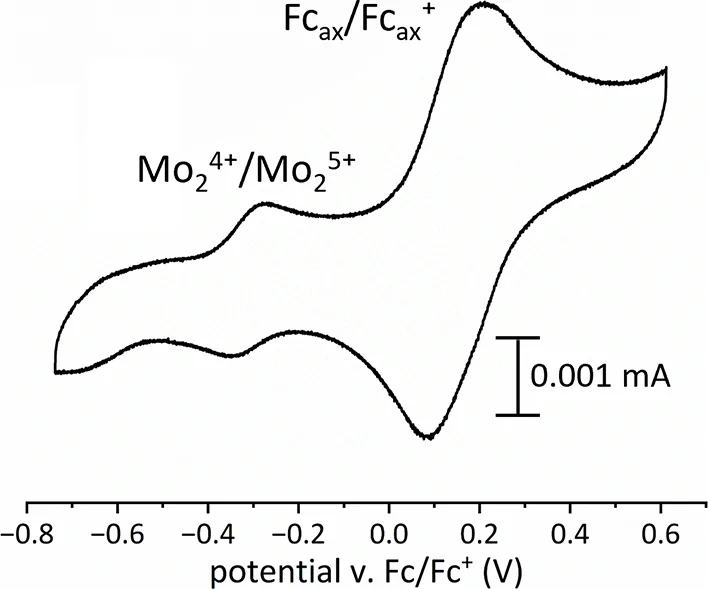
Cyclic voltammogram of **2** in Bu_4_NBF_4_·3PhCH_3_.

Within series **1**, we see a distinct effect of the para substituent of the axial benzoate ligand on the *E*_1/2_ of the Mo_2_^4+^/Mo_2_^5+^ couple. Specifically, the *E*_1/2_’s for series **1** span 40 mV and shift cathodically with increased electron-donating character of the axial ligand (Table 4). This trend is clearly illustrated in the bottom of Figure 5, which shows the linear free energy relationship between the Mo_2_^4+^/Mo_2_^5+^ redox couple and the sum of the Hammett parameters^15^ of the *para*-substituents of the O_2_CC_6_H_4_X axial ligands (2σ). We can attribute this effect to the destabilization of the Mo_2_ δ orbital upon the introduction of the electron-withdrawing substituent. This trend in *E*_1/2_’s also clearly demonstrates that, despite the structural and spectroscopic similarities of series **1**, the axial ligand clearly exerts a level of electronic “tuning” of the Mo≣Mo quadruple bond’s energy. From a more quantitative perspective, these linear free energy relationships take on the form of Δ*E*_1/2_ = *E*_1/2_(X) – *E*_1/2_(H) = ρ­(Σσ) = ρ­(2σ), where ρ is the slope and σ is the Hammett parameter for the ligand substituent R.^14,29,30^ Here, we note that the value of ρ determined from our measurements (0.026 V) is significantly smaller than that determined for the Mo_2_^4+^/Mo_2_^5+^ couple of the series of diarylformamidinate (DArF) supported Mo_2_ paddlewheel complexes (0.09 V), though we note that the latter series has eight substituents, two for each of the equatorial DArF ligands.^16,31^ Nevertheless, we may conclude that the electronic communication between Mo_2_ and the axial aryl groups in series **1** is not as significant as the communication between Mo_2_ and the equatorial aryl groups in the formamidinate series. This is a sensible result, since there is the potential for a direct orbital pathway for communication in the formamidinate case: the N–C­(H)–N π-system, with which the aryl groups will have some degree of conjugation, is of the proper symmetry to overlap with Mo_2_ orbitals of δ symmetry. For series **1**, the carboxylate is along the Mo–Mo axis and lacks orbitals with δ symmetry that can interact with the Mo_2_ frontier orbitals.

## Conclusions

This work builds on our previous studies of axial substitution of HEMAC complexes through the development of synthetic methods to prepare series **1** of Mo_2_Ni­(dpa)_4_(O_2_CC_6_H_4_X)_2_ HEMAC complexes, with X = CH_3_ (**1_CH**_**3**_), H (**1_H**), I (**1_I**), CN (**1_CN**), or CF_3_ (**1_CF**_**3**_), and HEAMC complex **2** with redox active ^–^O_2_CFc ligands. A combination of crystallographic characterization, electronic absorption spectroscopy, and SQUID magnetometry indicates that series **1** possesses similar structures in both the solid and solution state with high spin, *S* = 1 Ni^2+^ paramagnetic centers. However, electrochemical studies demonstrate that the Mo_2_^4+^/Mo_2_^5+^ redox couple depends on the electronic characteristics of the axial benzoate *para* substituent. This finding demonstrates that the electronics of the axial ligand can be used to modulate, albeit modestly, the electronics of the Mo≣Mo quadruple bond independently of substantial structural alterations in the proximity of the metal-atom chain.

## Supplementary Material



## Notes

Synthetic methods and experimental details of physical measurement techniques, crystallographic refinement details, additional figures, alternative models of magnetic data, and additional ^1^H and NMR spectra (PDF)

## References

[1] Krogman J. P., Thomas C. M. (2014). Metal-metal multiple bonding in C_3_–symmetric
bimetallic complexes of the first row transition metals. Chem. Commun..

[2] Chipman J. A., Berry J. F. (2020). Paramagnetic Metal-Metal
Bonded Heterometallic Complexes. Chem. Rev..

[3] Hua S.-A., Cheng M.-C., Chen C.-H., Peng S.-M. (2015). From Homonuclear
Metal String Complexes to Heteronuclear Metal String Complexes. Eur. J. Inorg. Chem..

[4] Georgiev V. P., Mohan P. J., DeBrincat D., McGrady J. E. (2013). Low-symmetry distortions
in Extended Metal Atom Chains (EMACs): Origins and consequences for
electron transport. Coord. Chem. Rev..

[5] Weng T., DeBrincat D., Arcisauskaite V., McGrady J. E. (2014). In search of structure–function
relationships in transition-metal based rectifiers. Inorg. Chem. Front..

[6] Tanaka Y., Kiguchi M., Akita M. (2017). Inorganic
and Organometallic Molecular
Wires for Single-Molecule Devices. Chem. Eur.
J..

[7] Chen P. J., Sigrist M., Horng E. C., Lin G. M., Lee G. H., Chen C. H., Peng S. M. (2017). A ligand design with a modified naphthyridylamide
for achieving the longest EMACs: the 1st single-molecule conductance
of an undeca-nickel metal string. Chem. Commun..

[8] Skipper H.
E., May C. V., Rheingold A. L., Doerrer L. H., Kamenetska M. (2021). Hard-Soft
Chemistry Design Principles for Predictive Assembly of Single Molecule-Metal
Junctions. J. Am. Chem. Soc..

[9] Nippe M., Berry J. F. (2007). Introducing a Metal–Metal
Multiply Bonded Group
as an “Axial Ligand” to Iron: Synthetic Design of a
Linear Cr–Cr···Fe Framework. J. Am. Chem. Soc..

[10] Wheaton A. M., Chipman J. A., Roy M. D., Berry J. F. (2022). Metal-Metal Bond
Umpolung in Heterometallic Extended Metal Atom Chains. Inorg. Chem..

[11] Turov Y., Berry J. F. (2012). Synthesis, characterization and thermal properties
of trimetallic N_3_–Cr≣Cr···
M–N_3_ azide complexes with M = Cr, Mn, Fe, and Co. Dalton Trans..

[12] Chipman J. A., Berry J. F. (2018). Facile Axial Ligand
Substitution in Linear Mo≣Mo-Ni
Complexes. Inorg. Chem..

[13] Nippe M., Turov Y., Berry J. F. (2011). Remote
effects of axial ligand substitution
in heterometallic Cr identical with Cr···M chains. Inorg. Chem..

[14] Zuman, P. . Substituent Effects In Organic Polarography; Springer, 1967.

[15] Hansch C., Leo A., Taft R. (1991). A survey of
Hammett substituent constants and resonance
and field parameters. Chem. Rev..

[16] Lin C., Protasiewicz J. D., Smith E. T., Ren T. (1996). Linear free energy
relationships in dinuclear compounds. 2. Inductive redox tuning via
remote substituents in quadruply bonded dimolybdenum compounds. Inorg. Chem..

[17] Bain G. A., Berry J. F. (2008). Diamagnetic corrections
and Pascal’s constants. J. Chem. Educ..

[18] Chilton N. F., Anderson R. P., Turner L. D., Soncini A., Murray K. S. (2013). PHI: a
powerful new program for the analysis of anisotropic monomeric and
exchange-coupled polynuclear d- and f-block complexes. J. Comput. Chem..

[19] Pickett C. (1985). A simple hydrocarbon
electrolyte: completing the electron-transfer series [Fe_4_S_4_(SPh)_4_]^1–/2–/3–/4–^. J. Chem. Soc., Chem. Commun..

[20] Fry A. J., Touster J. (1986). Voltammetry in (toluene)
3-tetrabutylammonium tetrafluoroborate,
a novel liquid hydrocarbon electrolyte. J. Orgo.
Chem..

[21] Cotton F. A., Daniels L. M., Murillo C. A., Timmons D. J., Wilkinson C. C. (2002). The extraordinary
ability of guanidinate derivatives to stabilize higher oxidation numbers
in dimetal units by modification of redox potentials: Structures of
Mo_2_
^5+^ and Mo_2_
^6+^ compounds. J. Am. Chem. Soc..

[22] Nippe M., Wang J., Bill E., Hope H., Dalal N. S., Berry J. F. (2010). Crystals in which
some metal atoms are more equal than
others: inequalities from crystal packing and their spectroscopic/magnetic
consequences. J. Am. Chem. Soc..

[23] Alvarez S. (2013). A cartography
of the van der Waals territories. Dalton Trans..

[24] Pyykkö P., Atsumi M. (2009). Molecular
single-bond covalent radii for elements 1–118. Chemistry.

[25] Nippe M., Victor E., Berry J. F. (2008). Do Metal-Metal Multiply-Bonded “Ligands”
Have a trans Influence? Structural and Magnetic Comparisons of Heterometallic
Cr≣Cr···Co and Mo≣Mo···Co
Interactions. Eur. J. Inorg. Chem..

[100] Spek A. L. (2015). PLATON SQUEEZE: a tool for the calculation
of the disordered
solvent contribution to the calculated structure factors. Acta. Crystallogr. Sect. C..

[26] Tsuzuki S., Fujii A. (2008). Nature and physical
origin of CH/π interaction: significant
difference from conventional hydrogen bonds. Phys. Chem. Chem. Phys..

[27] Chipman J. A., Berry J. F. (2018). Extraordinarily
Large Ferromagnetic Coupling (J ≥
150 cm^–1^) by Electron Delocalization in a Heterometallic
Mo≣Mo–Ni Chain Complex. Chem.—Eur.
J..

[28] Elschenbroich, C. ; Salzer, A. Organometallics; Wiley-VCH, 1992.

[29] Bender T.
P., Graham J. F., Duff J. M. (2001). Effect of substitution on the electrochemical
and xerographic properties of triarylamines: correlation to the Hammett
parameter of the substituent and calculated HOMO energy level. Chem. Mater..

[30] Heffner J. E., Raber J. C., Moe O. A., Wigal C. T. (1998). Using cyclic
voltammetry and molecular modeling to determine substituent effects
in the one-electron reduction of benzoquinones. J. Chem. Educ..

[31] Lin C., Protasiewicz J. D., Smith E. T., Ren T. (1995). Redox tuning of the
dimolybdenum compounds at the ligand periphery: a direct correlation
with the Hammett constant of the substituents. J. Chem. Soc., Chem. Commun..

