# Supplementary material for: Heterometallic Extended Metal Atom Chain Complexes with Carboxylate (O2CX) Axial Ligands
Source: Inorg Chem. 2026 Aug 10;65(33):19275–83. doi: 10.1021/acs.inorgchem.6c02287 (PMC13508997; doi:10.1021/acs.inorgchem.6c02287)
Supplement: Supplementary file 1 [file ic6c02287_si_001.pdf]

Supporting information for

**Heterometallic extended metal atom chain complexes with carboxylate ($O_2CX$) axial ligands**

Amelia M. Wheaton, Milton Acosta, John F. Berry*

Department of Chemistry, University of Wisconsin – Madison, 1101 University Ave., Madison, Wisconsin 53706, United States

*Email: berry@chem.wisc.edu

**Contents:**

| **Description** | **Page** |
|---|---|
| **Figures S1 – S12: NMR Data** | **S2-S13** |
| **Tables S1 & S2: Crystallographic Data** | **S14-S15** |
| **Crystallographic Info, with Figures S13–S26** | **S16-S28** |
| **Magnetic Susceptibility Comments** | **S29** |
| **Table S3** | **S30** |
| **Figure S27** | **S39** |
| **Table S4** | **S30** |
| **Figures S28 and S29** | **S31** |
| **References** | **S32** |

**Figure S1**: $^{1}$HNMR spectra of $[NEt_4][O_2CC_6H_4CH_3]$ in $CD_3CN$

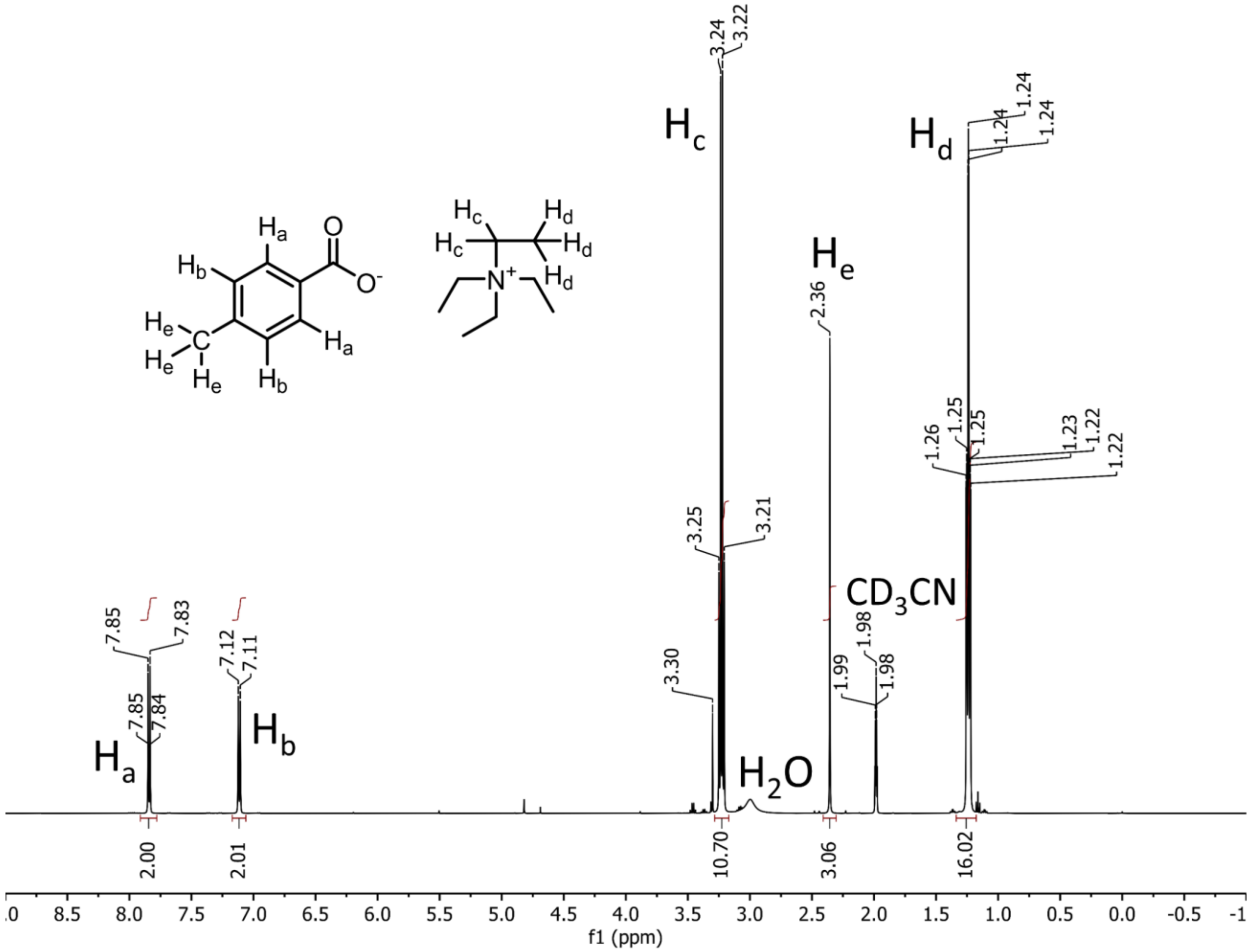

**Figure S2**: $^{13}$CNMR spectra of $[NEt_4][O_2CC_6H_4CH_3]$ in $CD_3CN$

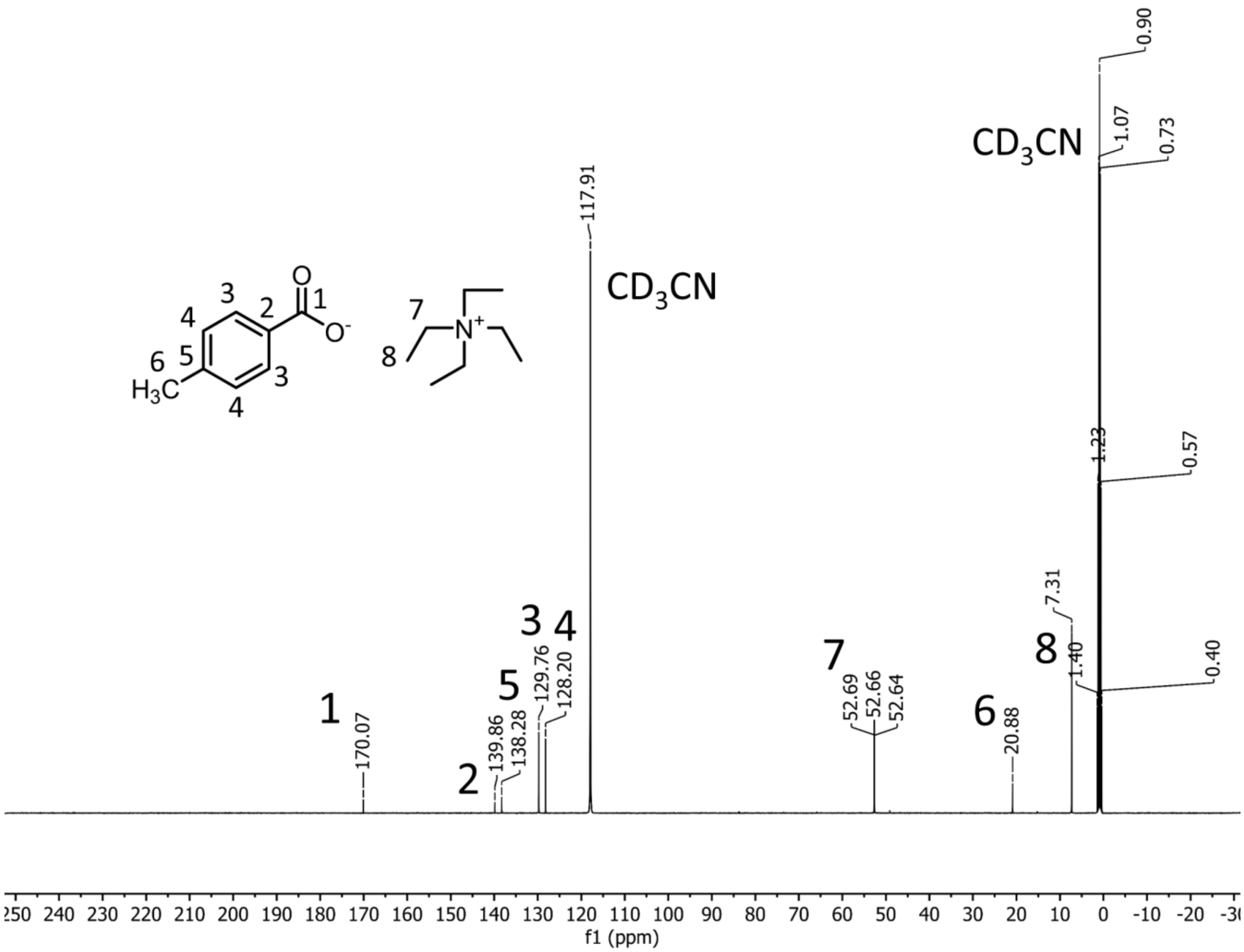

**Figure S3**: $^{1}H$ NMR spectra of $[NEt_4][O_2CC_6H_5]$ in $CD_3CN$

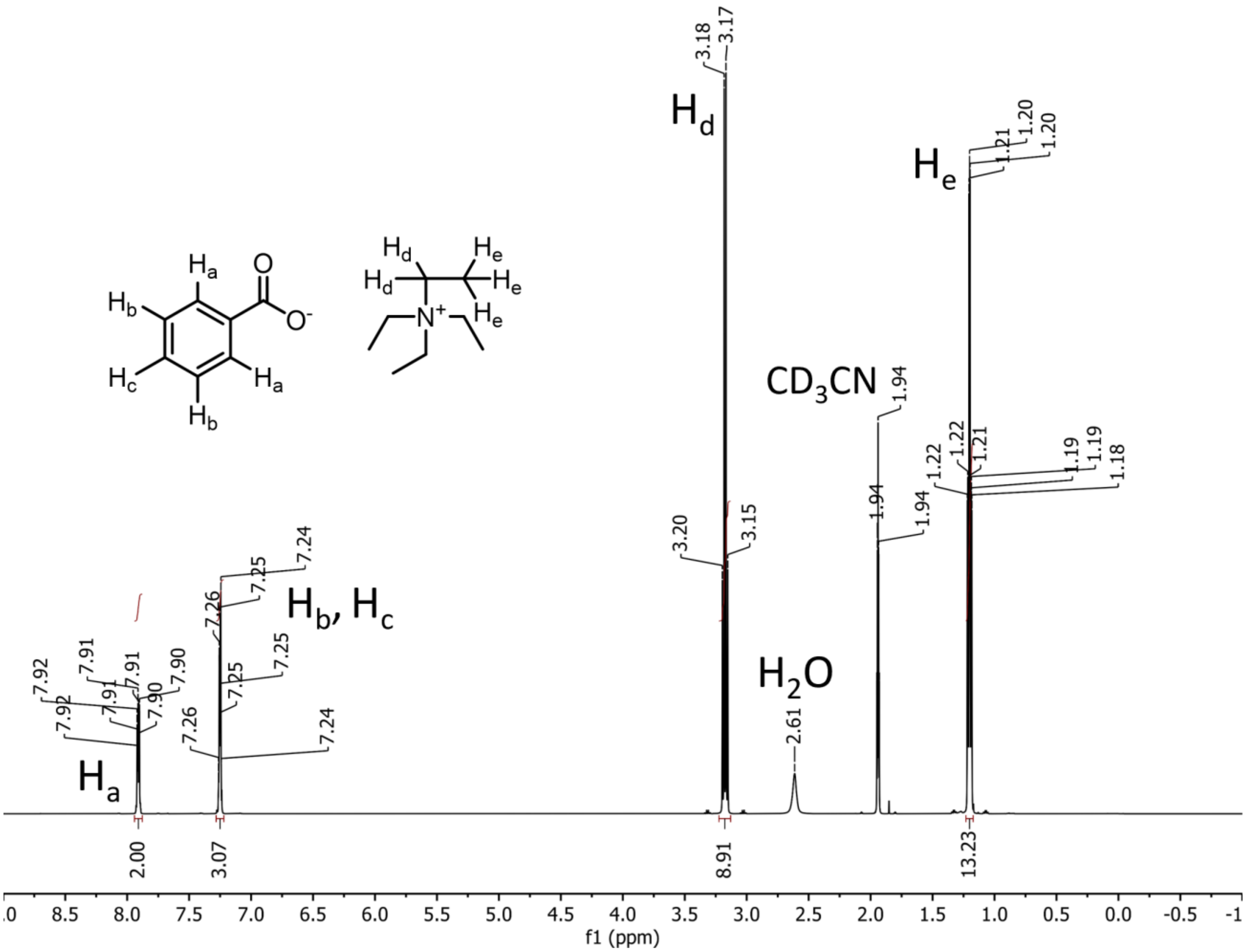

**Figure S4**: $^{13}$CNMR spectra of $[NEt_4][O_2CC_6H_5]$ in $CD_3CN$

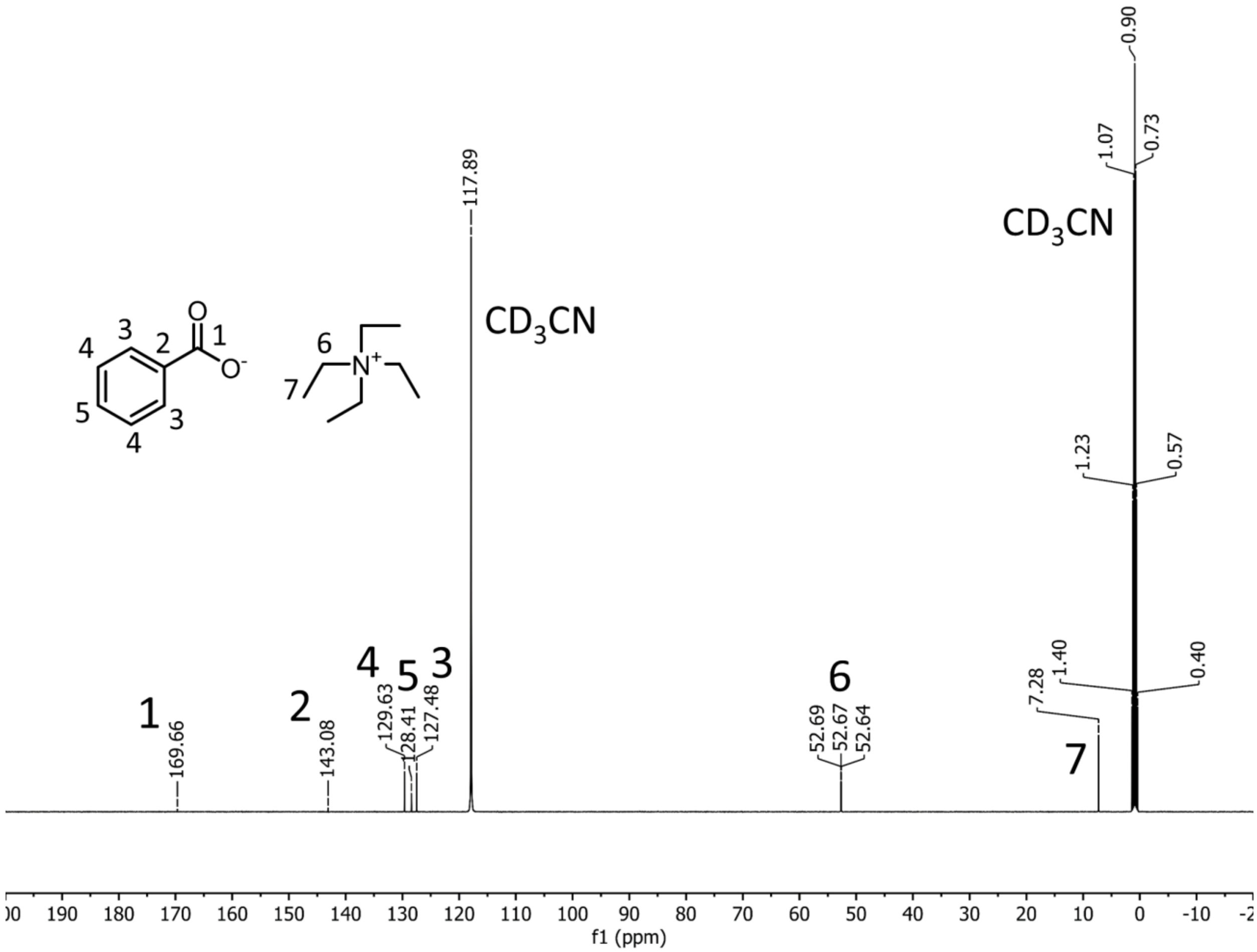

**Figure S5**: $^{1}$HNMR spectra of $[NEt_4][O_2CC_6H_4I]$ in $CD_3CN$

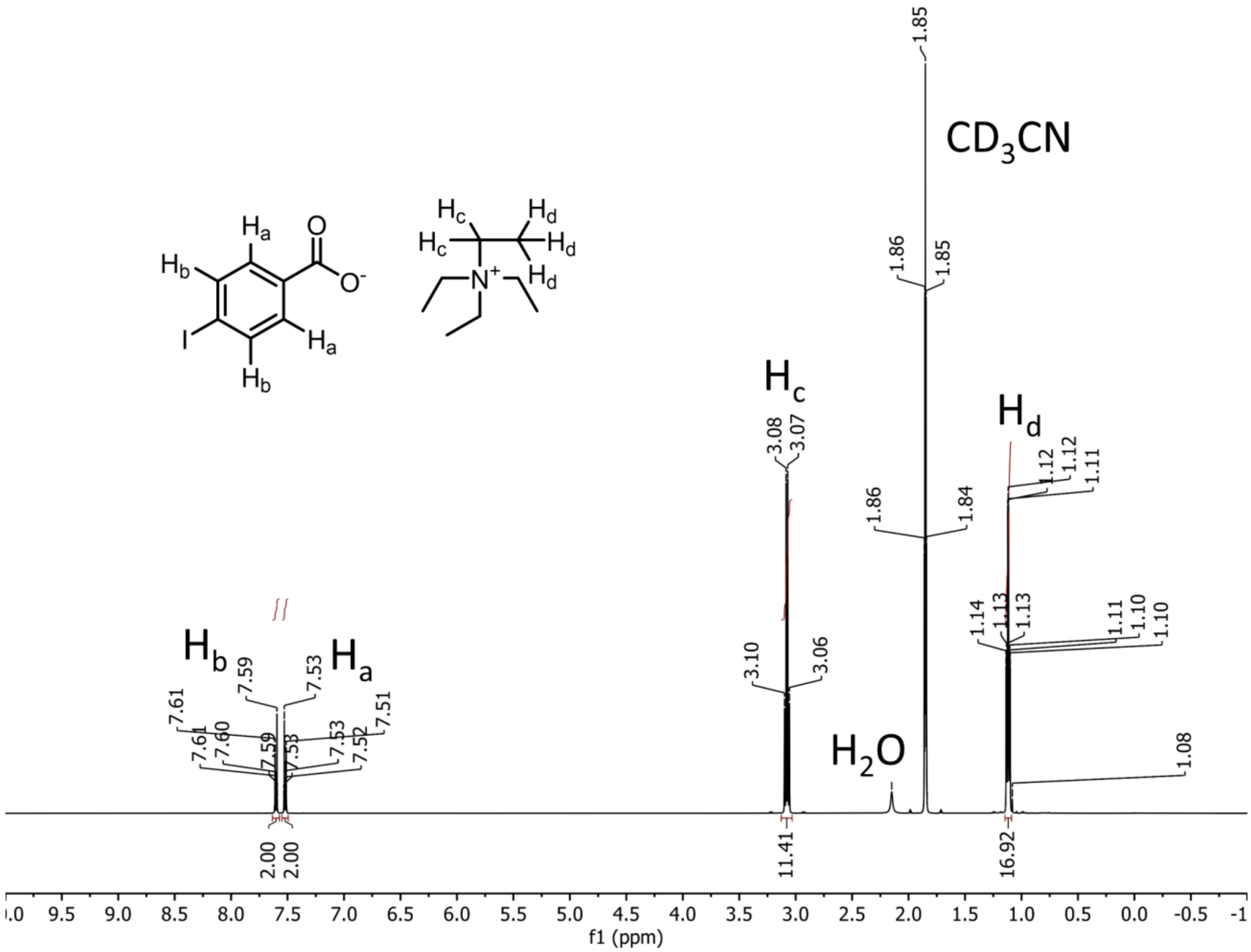

**Figure S6**: $^{13}$CNMR spectra of $[NEt_4][O_2CC_6H_4I]$ in $CD_3CN$

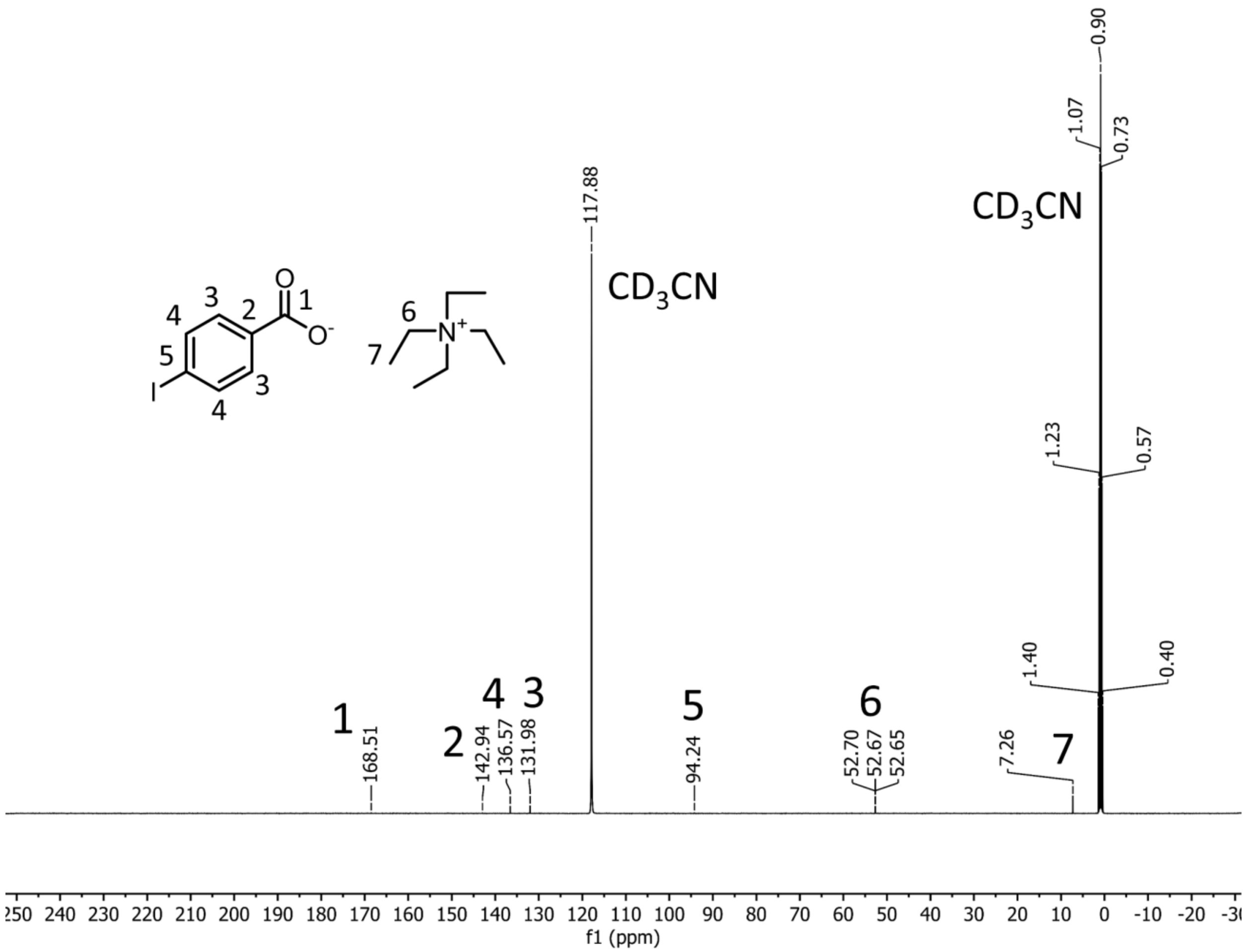

**Figure S7**: $^{1}$HNMR spectra of $[NEt_4][O_2CC_6H_4CF_3]$ in $CD_3CN$

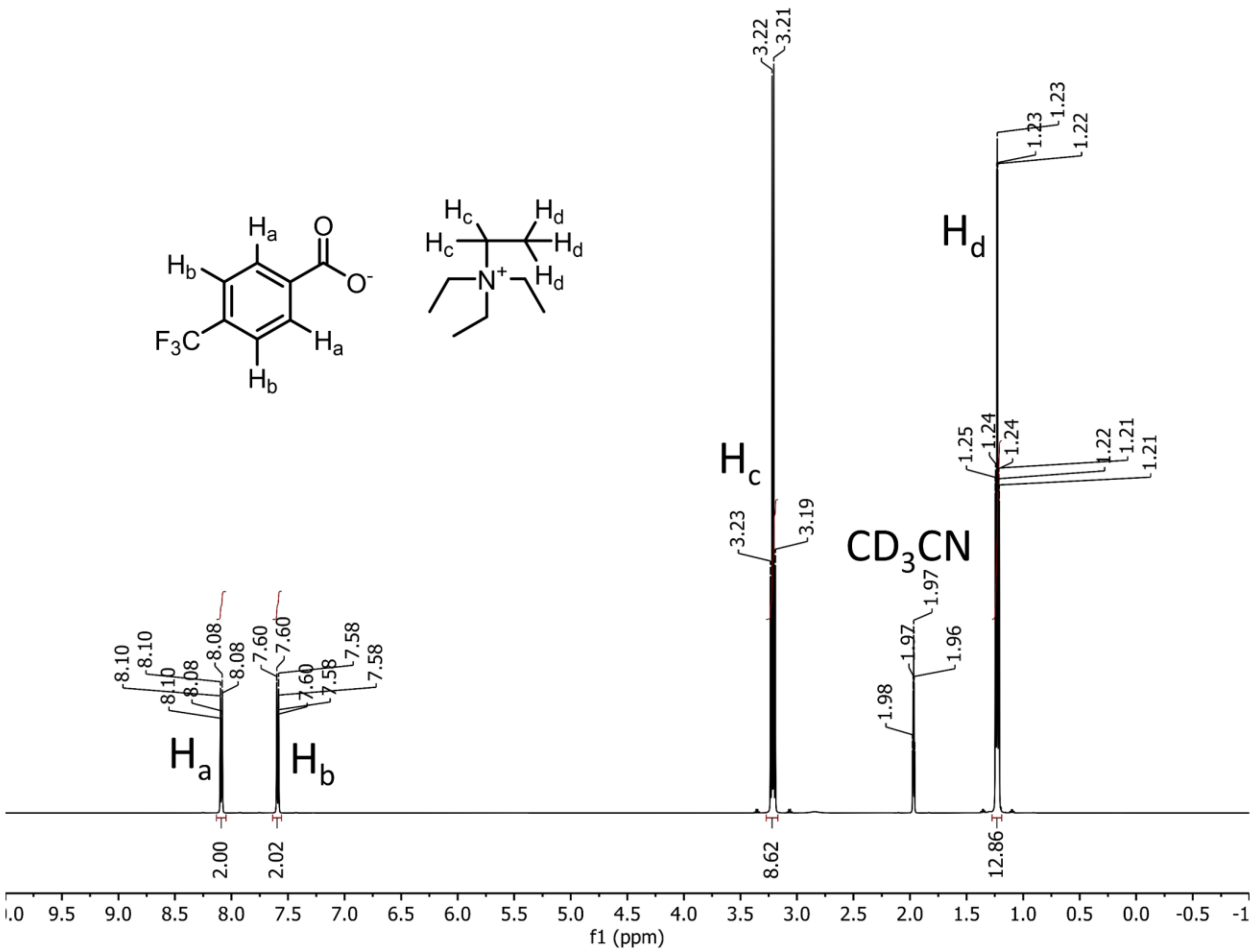

**Figure S8**: $^{13}$CNMR spectra of $[NEt_4][O_2CC_6H_4CF_3]$ in $CD_3CN$

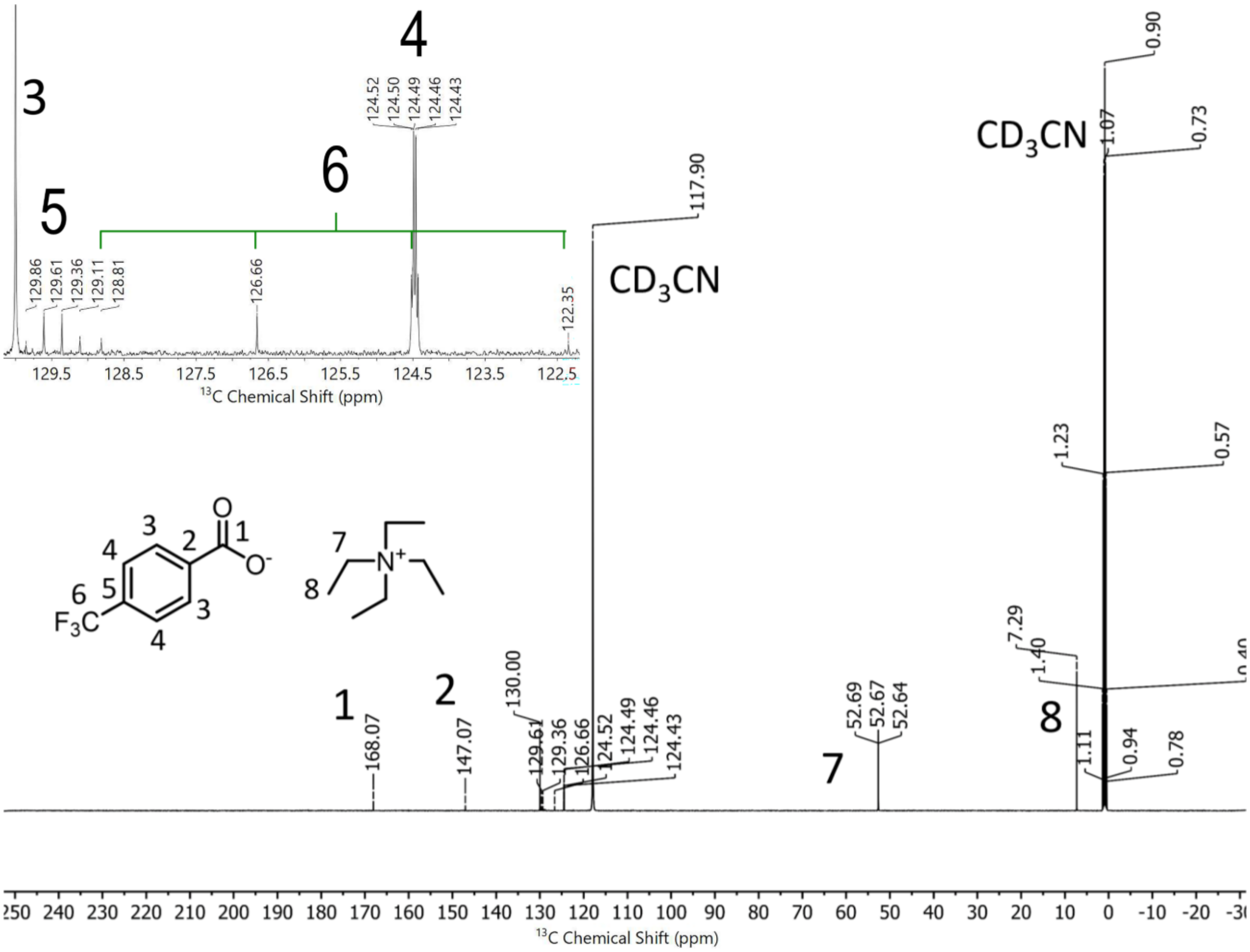

**Figure S9**. $^{1}$HNMR spectra of $[NEt_4][O_2CC_6H_4CN]$ in $CD_3CN$

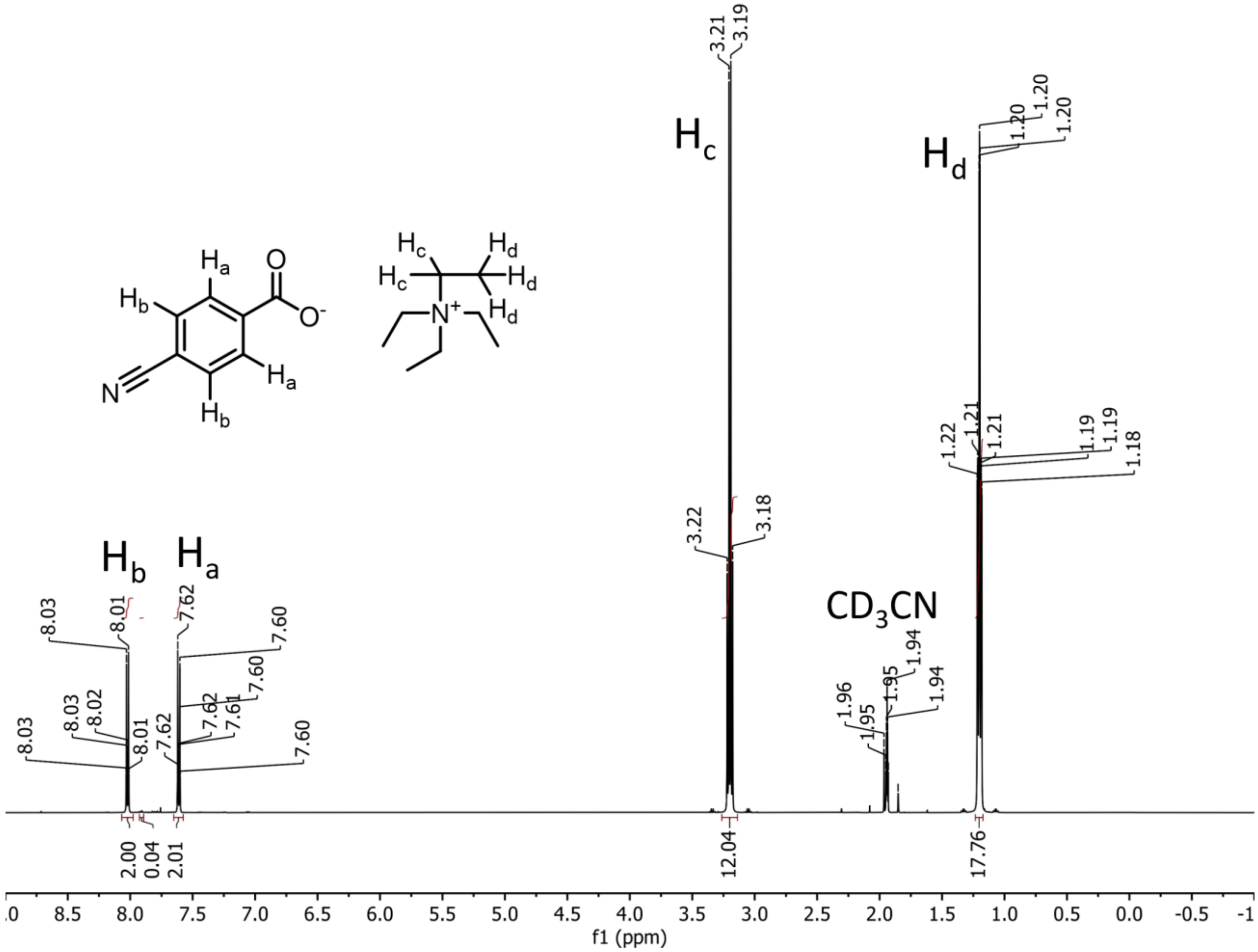

**Figure S10**. $^{13}$CNMR spectra of $[NEt_4][O_2CC_6H_4CN]$ in $CD_3CN$

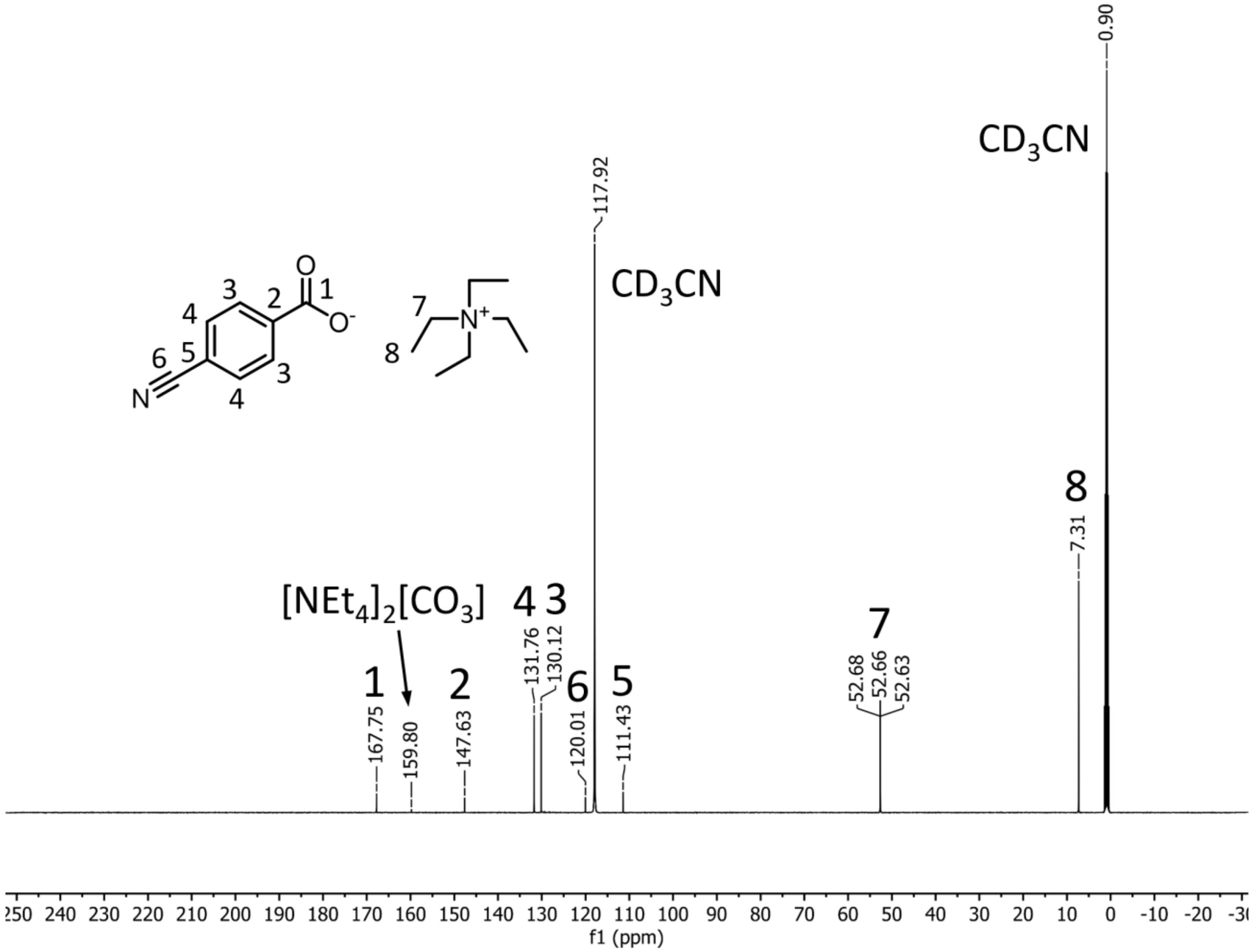

**Figure S11**. $^{13}$HNMR spectra of $[NEt_4][O_2CFc]$ in $CD_3CN$

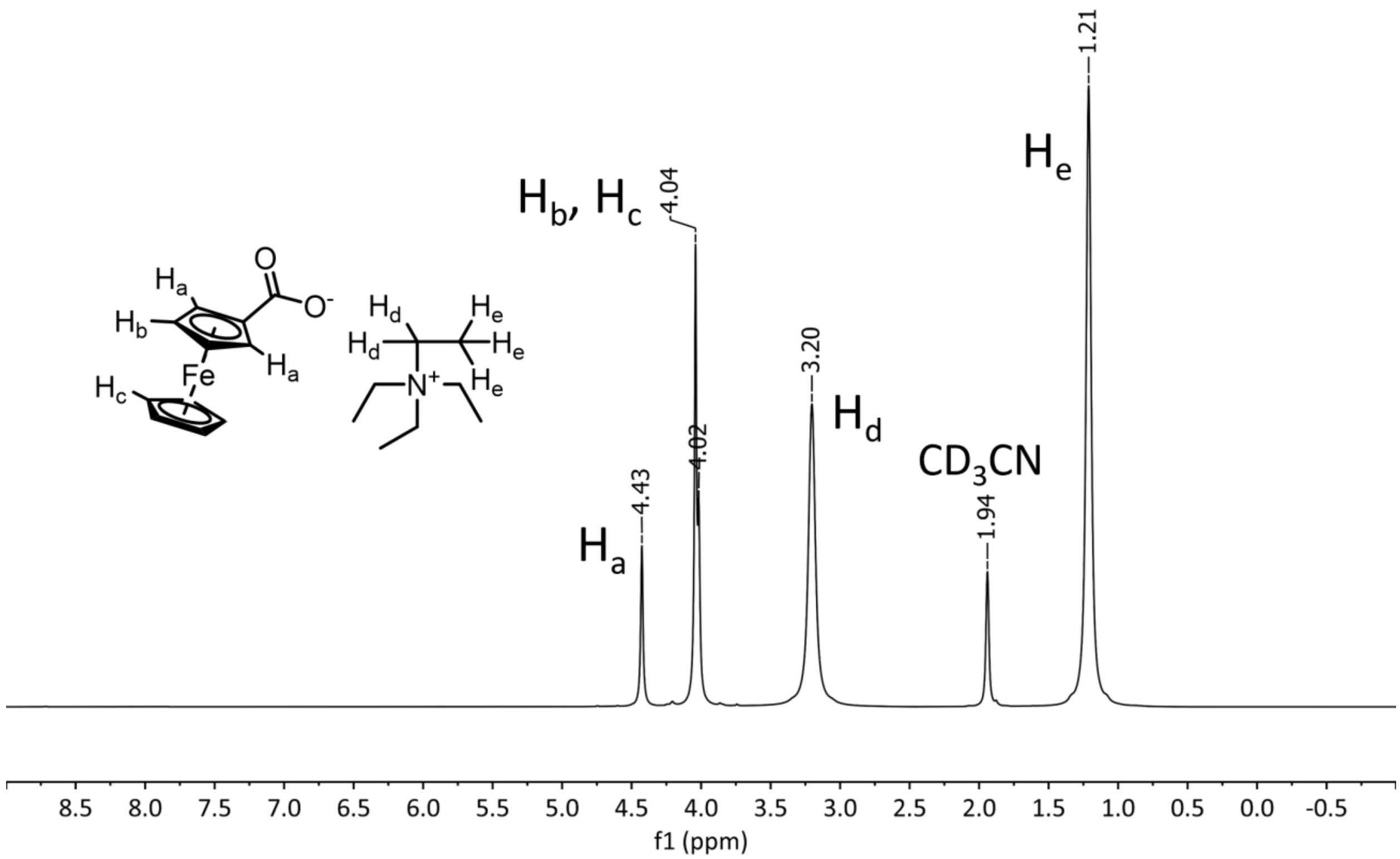

**Figure S12**. $^{13}$CNMR spectra of $[NEt_4][O_2CFc]$ in $CD_3CN$

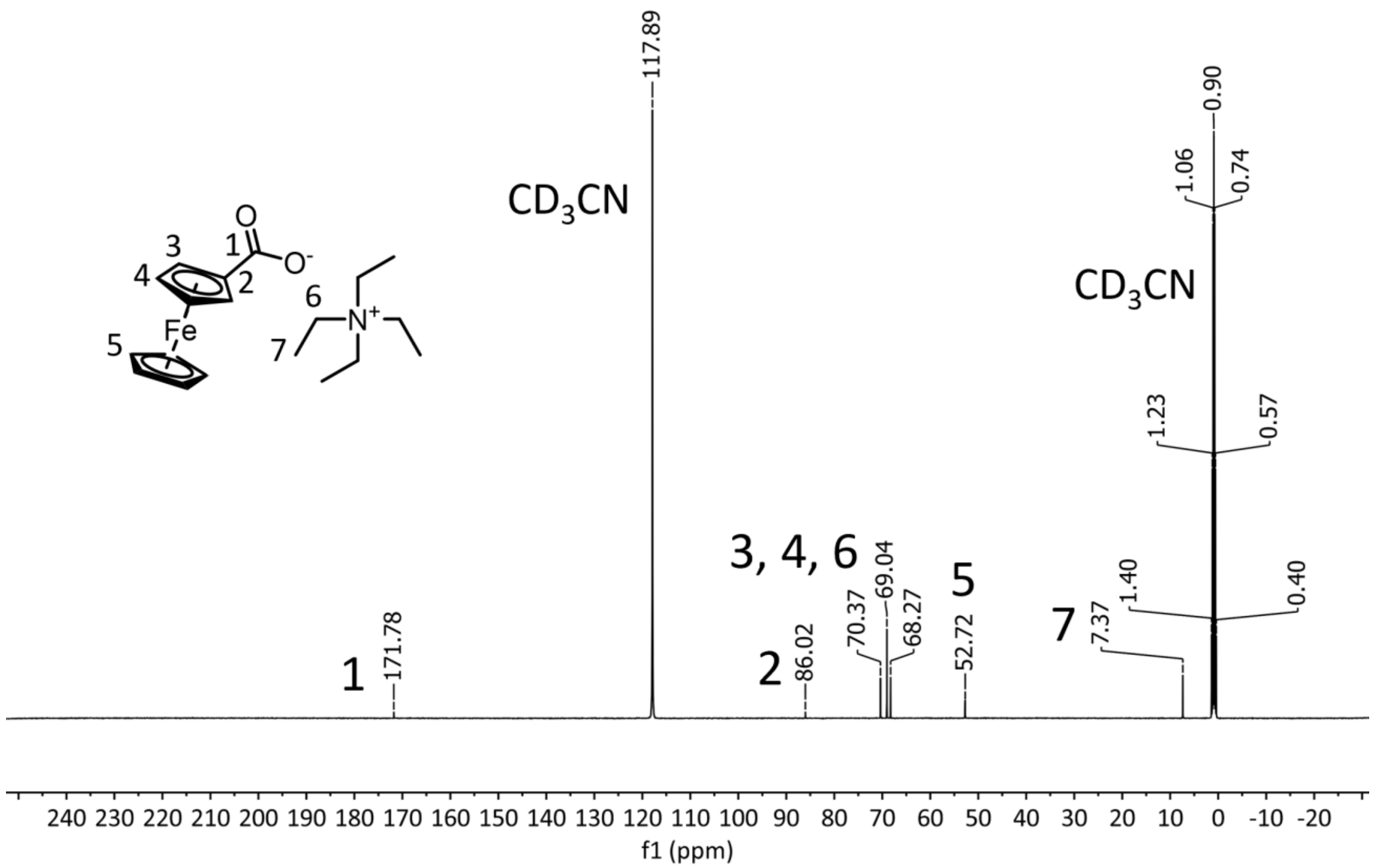

**Table S1.** Crystal data for **1_CH$_3$** · $PhCH_3$, **1_H**·3 $C_7H_8$, and **1_CF$_3$** ·2.87 $C_7H_8$

| | **1_CH$_3$** · $PhCH_3$ | **1_H** · 3 $PhCH_3$ | **1_CF$_3$**· ·2.87 $PhCH_3$ |
|---|---|---|---|
| Empirical formula | $Mo_2Ni(C_{10}H_8N_3)_4(O_2CC_6H_5)_2$ · 2 $PhCH_3$ | $Mo_2Ni(C_{10}H_8N_3)_4(O_2CC_6H_5)_2$ · 3 $PhCH_3$ | $Mo_2Ni(C_{10}H_8N_3)_4(O_2CC_6H_4CF_3)_2$ · 2.87 $PhCH_3$ |
| Crystal system | tetragonal | orthorhombic | monoclinic |
| Space group | $P\bar{4}2_1c$ | $Pna2_1$ | $P2_1/c$ |
| $a$/Å | 14.2634(16) | 18.774(2) | 13.1886(16) |
| $b$/Å | 14.2634(16) | 13.0654(17) | 28.537(3) |
| $c$/Å | 31.550(3) | 26.298(3) | 18.383(3) |
| $\alpha$/° | 90 | 90 | 90 |
| $\beta$/° | 90 | 90 | 96.381(7) |
| $\gamma$/° | 90 | 90 | 90 |
| Volume/Å$^3$ | 6418.8(16) | 6450.4(14) | 6875.8(15) |
| Z | 4 | 4 | 4 |
| $\rho_{calc}$ g/cm$^3$ | 1.243 | 1.485 | 1.514 |
| $R_1$$^a$, $wR_2$$^b$ [I>=2σ (I)] | 0.0504, 0.1305 | 0.0493; 0.1087 | 0.0662, 0.1552 |
| $R_1$$^a$, $wR_2$$^b$ [all data] | 0.0588, 0.1415 | 0.0538; 0.1113 | 0.0946, 0.1702 |

$^a$ $R_1 = \sum||F_o| - |F_c||/\sum|F_o|$. $^b$$wR_2= [\sum[w(F_o^2 - F_c^2)^2]/\sum[w(F_o^2)^2]]^{1/2}$, $w = 1/\sigma^2(F_o^2) + (aP)^2 + bP$, where $P = [max(0 \text{ or } F_o^2) + 2(F_c^2)]/3$

**Table S2.** Crystal data for **4** ($Mo_2Ni(dpa)_4Cl_2 \cdot 2\ CH_2Cl_2$), $Mo_2(dpa)_4(OAc)$ and $[Mo_2(C_{10}H_8N_3)_4][O_3SCF_3]$

| | | | |
|---|---|---|---|
| Empirical formula | $Mo_2Ni(dpa)_4Cl_2 \cdot 2\ CH_2Cl_2$ | $Mo_2(dpa)_3(O_2CCH_3)$ | $[Mo_2(C_{10}H_8N_3)_4][O_3SCF_3]$ |
| Crystal system | monoclinic | triclinic | monoclinic |
| Space group | $C2/c$ | $P\bar{1}$ | $P2_1/n$ |
| $a$/Å | 18.875(6) | 10.318(3) | 16.546(6) |
| $b$/Å | 16.069(6) | 12.034(3) | 8.820(3) |
| $c$/Å | 15.815(6) | 12.189(4) | 27.959(8) |
| $\alpha$/° | 90 | 95.067(8) | 90 |
| $\beta$/° | 112.242(7) | 95.638(9) | 104.673(8) |
| $\gamma$/° | 90 | 102.829(13) | 90 |
| Volume/Å$^3$ | 4440(3) | 1459.1(7) | 3947(2) |
| Z | 4 | 2 | 4 |
| $\rho_{calc}$ g/cm$^3$ | 1.754 | 1.733 | 1.719 |
| $R_1{}^a$, $wR_2{}^b$ [I>=2σ (I)] | 0.0280, 0.0686 | 0.0236; 0.0611 | 0.0371; 0.0933 |
| $R_1{}^a$, $wR_2{}^b$ [all data] | 0.0385, 0.0743 | 0.0259; 0.0623 | 0.0466; 0.0974 |

[a] $R_1 = \sum||F_o| - |F_c||/\sum|F_o|$. [b] $wR_2 = [\sum[w(F_o{}^2 - F_c{}^2)^2]/\sum[w(F_o{}^2)^2]]^{1/2}$, $w = 1/\sigma^2(F_o{}^2) + (aP)^2 + bP$, where $P = [\max(0 \text{ or } F_o{}^2) + 2(F_c{}^2)]/3$

**Crystallographic methods and refinement details**.

Crystals were mounted on the tip of a MiTeGen MicroMount under oil under an atmosphere of Ar, transferred to the goniometer head under a stream of cold nitrogen at 100(1) K, and centered in the X-ray beam. Data collection for **1_CH₃**, **1_H**, and **1_CF₃** were performed on a Bruker D8 VENTURE PhotonIII four-circle diffractometer with Cu Kα (λ = 1.54178 Å) radiation with a detector to crystal distance of 4.0 cm. Data collection for $Mo_2Ni(dpa)_4Cl_2$, $Mo_2(dpa)_3(O_2CCH_3)$, and $[Mo_2(dpa)_4][O_3SCF_3]$ were performed on a Bruker Quazar SMART APEXII diffractometer with Mo $K_\alpha$ (λ = 0.71073 Å) radiation with a detector distance of 4.96 cm. The data were collected using a routine to survey the entire sphere of reciprocal space and were indexed by the SMART program.[1] The structures were solved using direct methods[2] and refined by least squares refinement on $F^2$ followed by difference Fourier synthesis.[3] All hydrogen atoms were included in the final structure factor calculation at idealized positions and were allowed to ride on the neighboring atoms with relative isotropic displacement coefficients. Additional molecular drawings and details about the crystallographic models for **$1\_CH_3$** · $C_7H_8$, **1_H**·3 $C_7H_8$, **$1\_CF_3$** · 2.87 $PhCH_3$, $Mo_2Ni(dpa)_4Cl_2$, $Mo_2(dpa)_3(O_2CCH_3)$, and $[Mo_2(dpa)_4][O_3SCF_3]$ are described below:

**$1\_CH_3$** · $C_7H_8$: The compound crystallizes in space group $P\bar{4}2_1c$ (#114), which is noncentrosymmetric but also neither chiral nor polar. The HEMAC complex resides on a crystallographic two-fold axis perpendicular to the direction of the metal atom chain, and as such only one half of the complex is symmetry independent. The symmetry independent metal atoms Mo1, Mo2, and Ni1, and the central amide N atoms of the dipyridylamine ligands (N2 and N5) are disordered across the two-fold axis. The disordered atoms were constrained to have 50 % occupancies. Atoms pairs Ni1/Mo2 and N2/N5 were constrained to have equivalent anisotropic displacement parameters. The atom pairs N1–Ni1/N6–Ni1 and N1–Mo2/N6–Mo2 were restrained to have similar 1,2 distances.

There is one molecule of toluene present in the asymmetric unit that is disordered over at least two positions. Bond length constraints were applied to model the disorder components, but the resulting isotropic displacement coefficients suggested the molecule was mobile. Option SQUEEZE of the program PLATON was used to correct the diffraction data for diffuse scattering effects and to identify the solvate molecule.[4] PLATON calculated the upper limit of volume that can be occupied by the solvent to be 1937 $Å^3$, or 30 % of the unit cell volume. The program calculated 59 electrons in the unit cell for the diffuse species. This approximately corresponds to one molecule of toluene in the asymmetric unit (50 electrons). All derived results are based on the known contents of the unit cell and no data are provided for the diffusely scattering species. However, we report the toluene molecule in the molecular formula for the structure.

The absolute configuration of the molecule was unambiguously determined by anomalous dispersion effects; Flack x = 0.02(4) %.

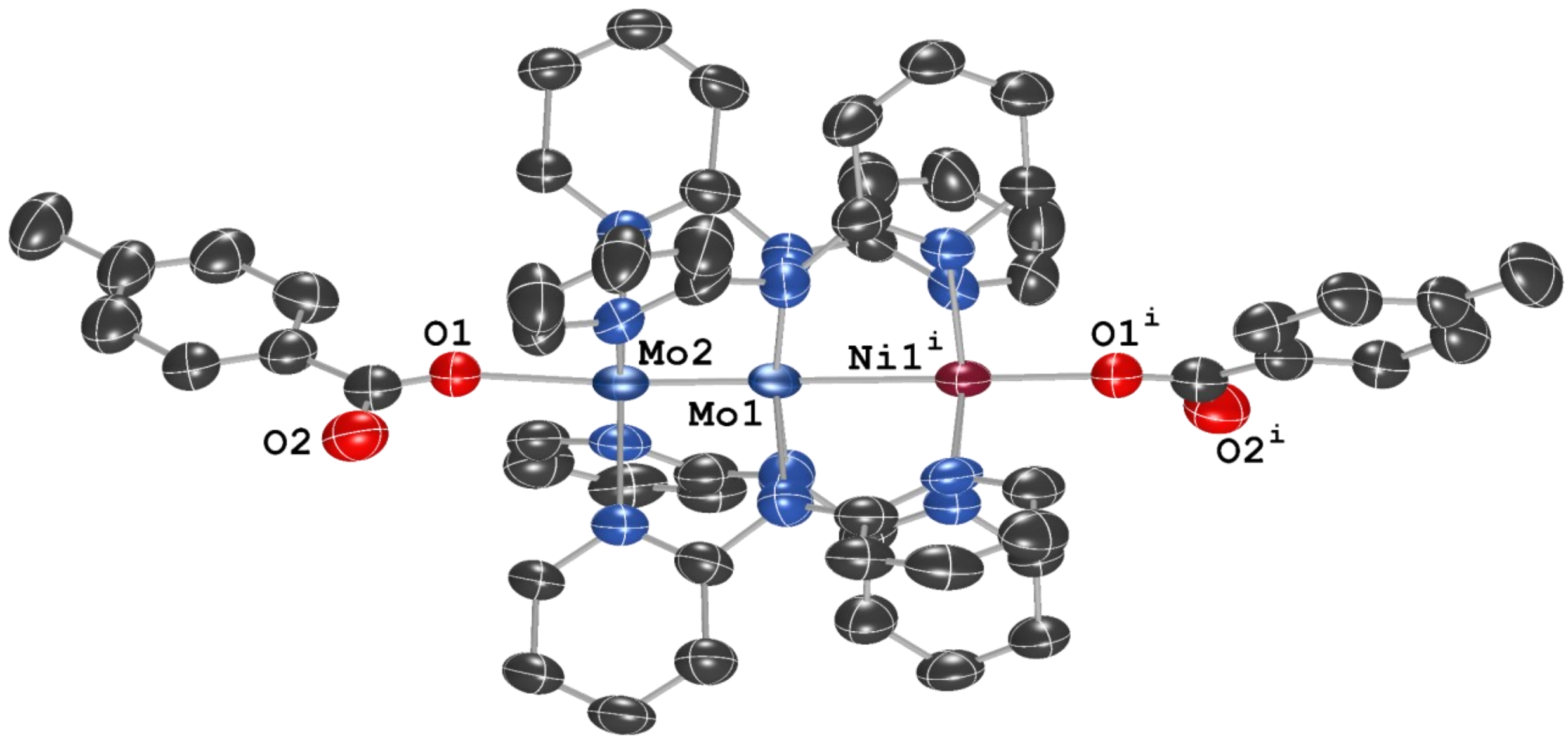


**Figure S13**. A molecular drawing of **1_CH3** shown with 50% probability ellipsoids and selected atom labels. All H atoms and minor disorder components are omitted for clarity. [Symmetry code: (i) 1−*x*, 1−*y*, *z*.]

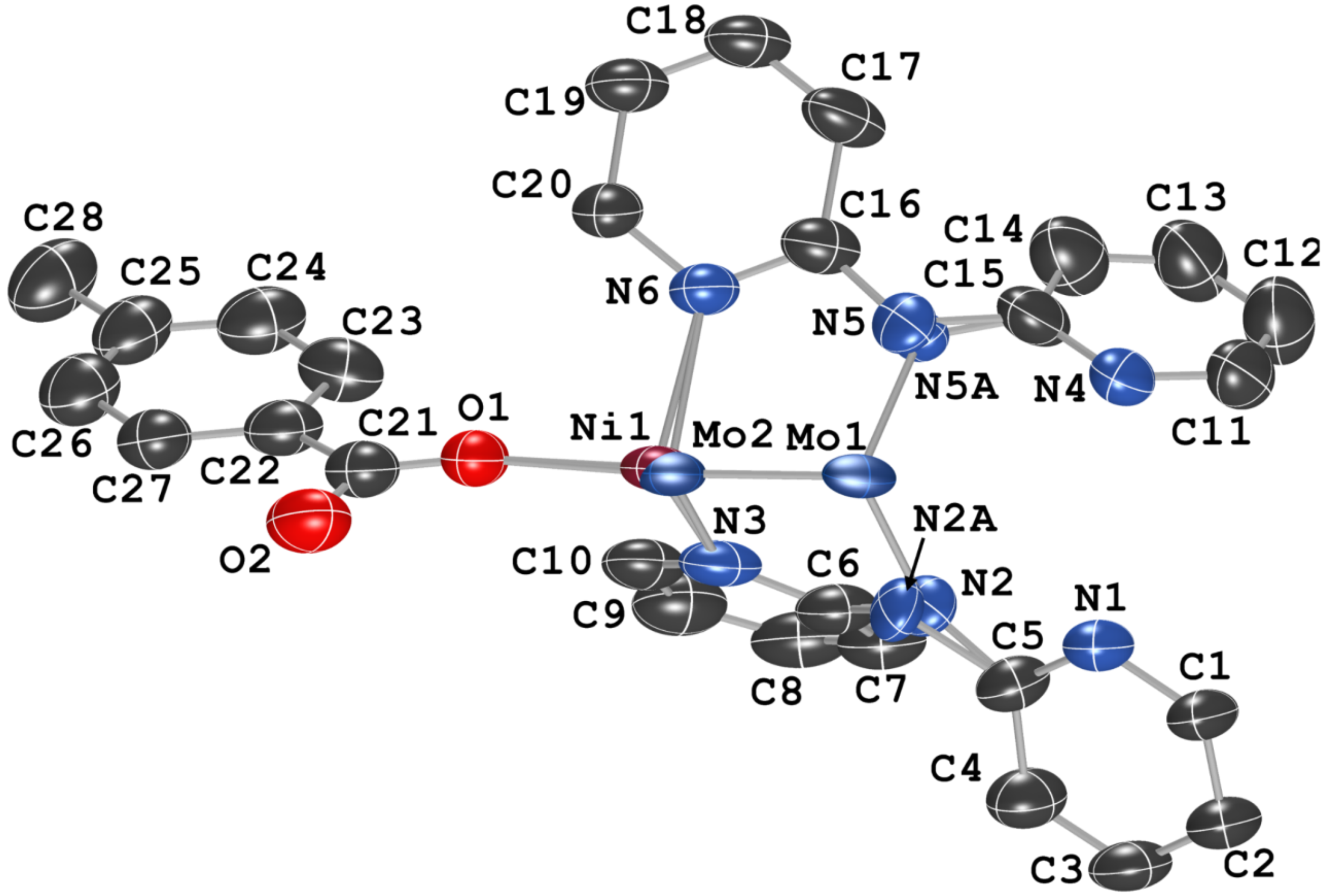


**Figure S14**. A molecular drawing of the symmetry-independent unit of **1_CH3** showing both components of the metal-atom disorder. All atoms are shown with 50% probability ellipsoids; all H atoms are omitted for clarity.

**1_H**· 3 $C_7H_8$: The compound crystallizes in the noncentrosymmetric and polar space group *Pna*$2_1$ (#33). The molecule lies on a general position, but the metal atoms Mo1, Mo2, and Ni1, the central amide N atoms of the dipyridylamine ligands N2, N5, N8, and N11, and the N3 and N4 pyridyl rings of the dpa ligand are disordered over two positions with a major component occupancy of 70.9(6) %. The disordered metal atoms were refined with atomic displacement parameter constraints. The N and C atoms in the minor disorder component were refined isotropically, and the N and C atoms in both disorder components were refined with atomic displacement parameters and geometric restraints. For Mo1 and Mo2, the Mo–N distances of the major and minor disordered components were restrained to be similar.

The asymmetric unit also contains three molecules of toluene, and two of the solvate molecules (corresponding to the C55 and C62 solvates) are disordered over two positions with major component occupancies of 58.41 and 55.63 %. These occupancies were initially refined, then fixed. The disordered molecules were refined with idealized geometries.[5] All three molecules were refined with atomic displacement parameter constraints and restraints.

The structure crystallized as an inversion twin, with a 35(2) % contribution from the minor component.

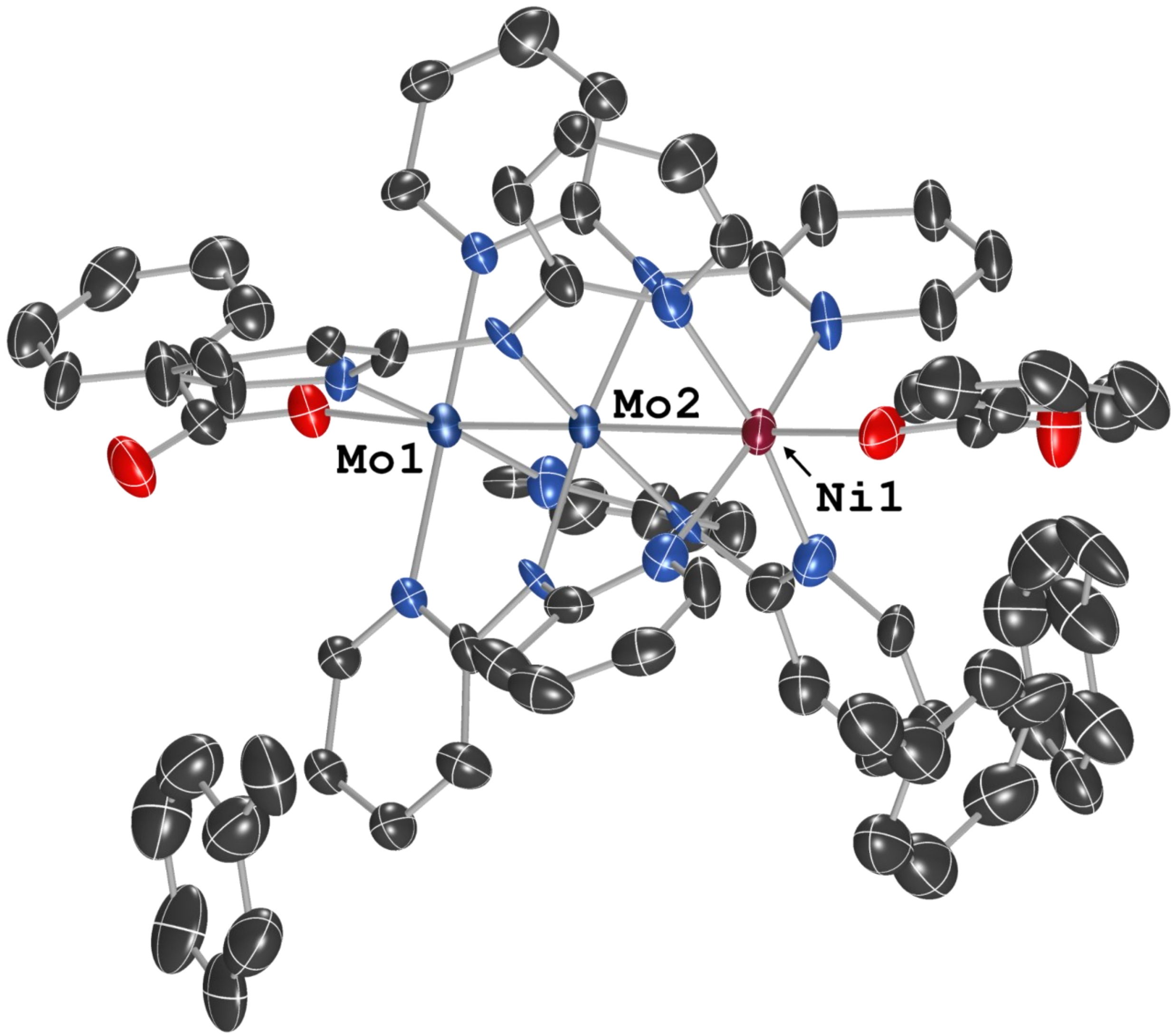


**Figure S15**. A molecular drawing of **1_H** shown with 50% probability ellipsoids and selected atom labels. All H atoms and minor disorder components are omitted for clarity.

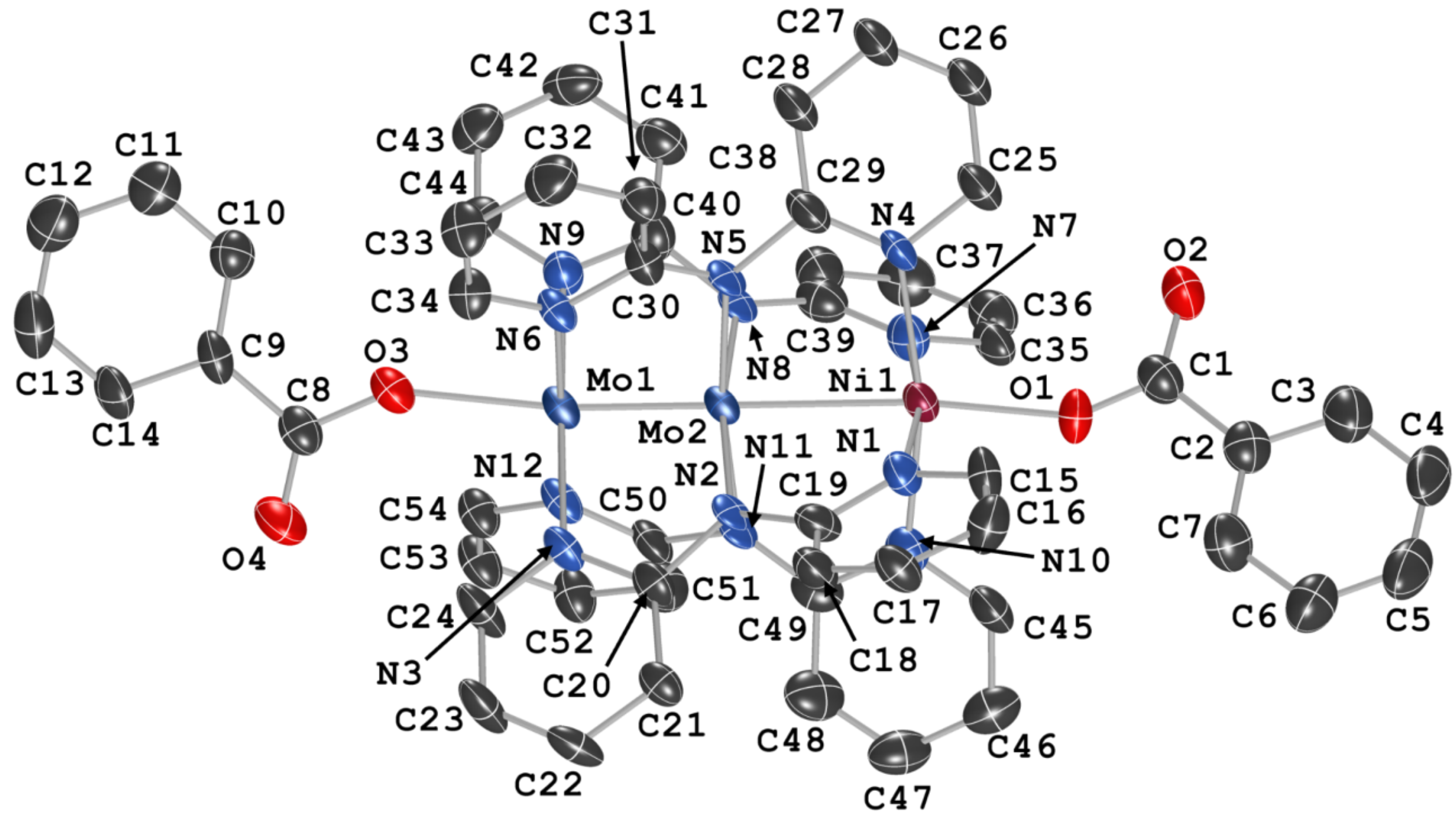


**Figure S16.** A molecular drawing of the HEMAC complex in **1_H**. All atoms are shown with 50% probability ellipsoids; all H atoms, minor disorder components, and solvent molecules are omitted for clarity.

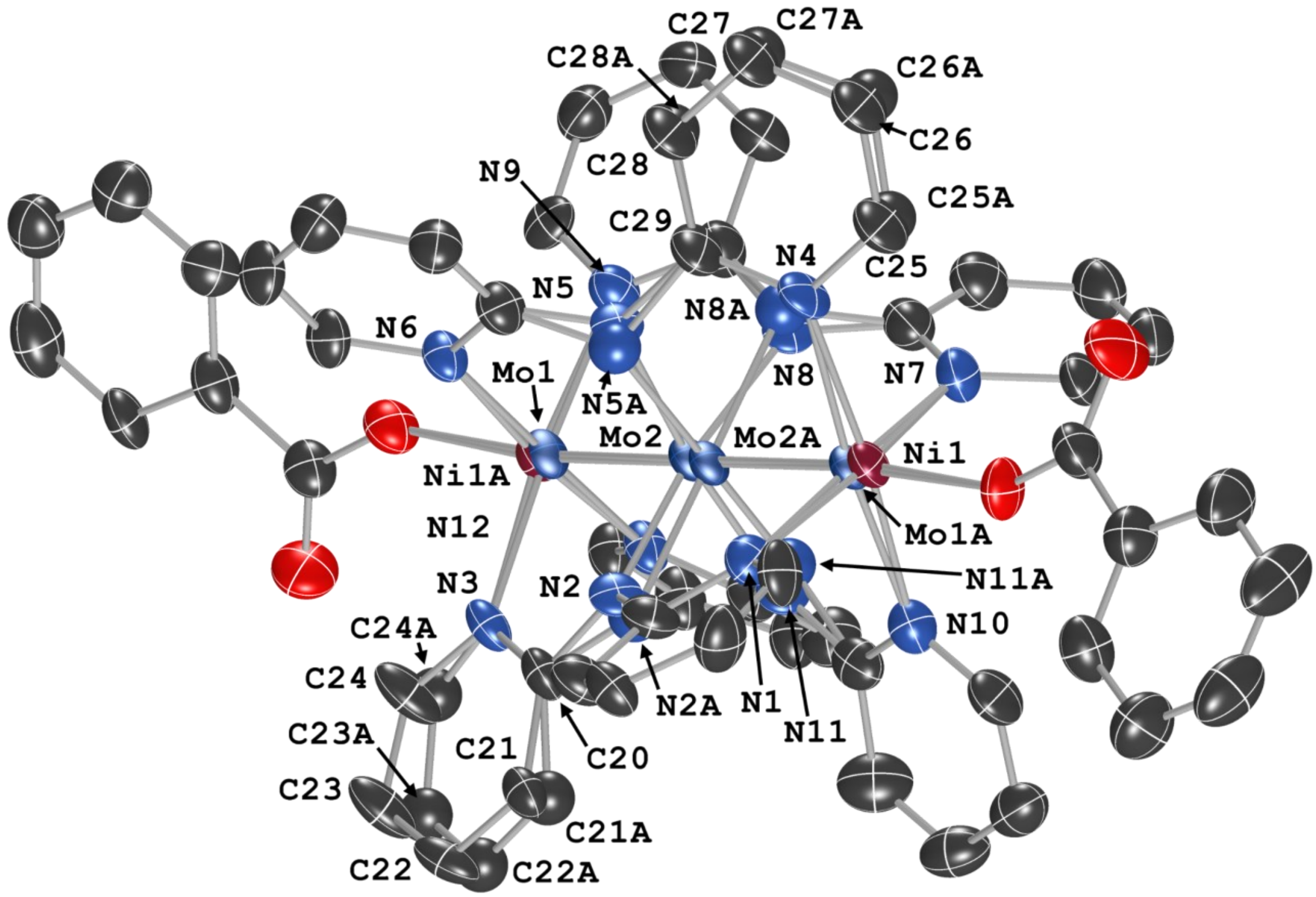


**Figure S17**. A molecular drawing of the HEMAC complex in **1_H** with selected atom labels and both metal atom disorder components shown. All atoms are shown with 50% probability ellipsoids; all H atoms and solvent molecules are omitted for clarity.

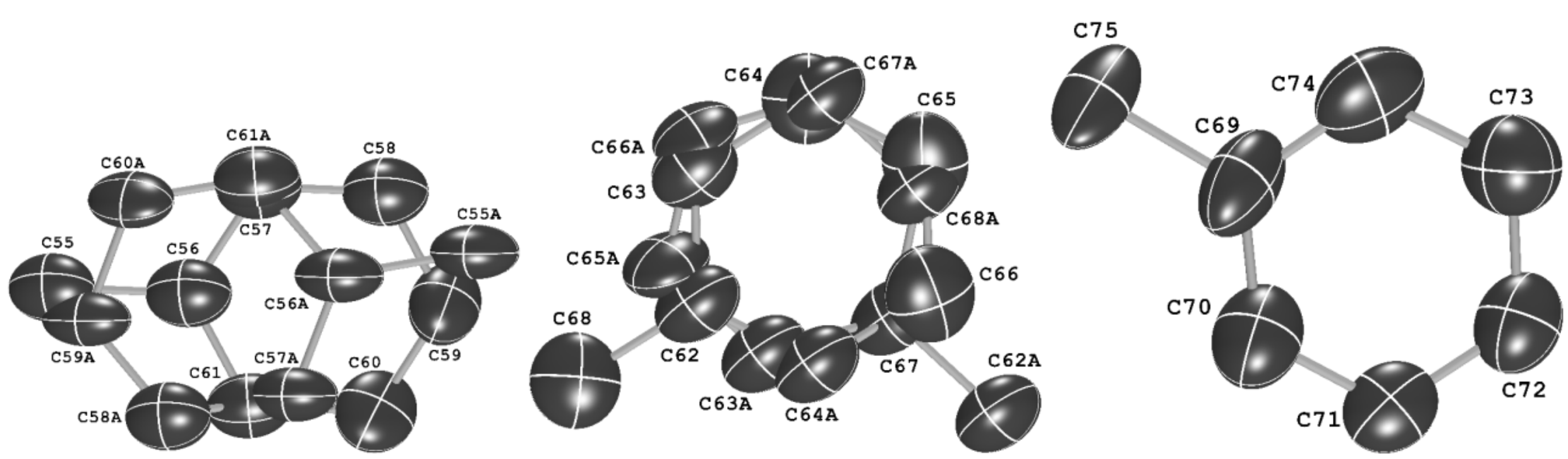


**Figure S18**. A molecular drawing of first, second, and third symmetry-independent molecules of solvate toluene in **1_H**; all H atoms are omitted for clarity.

**1_CF3** · 2.87 $C_7H_8$ : The molecule crystallizes on a general position in the centrosymmetric space group $P2_1/c$ (#14). The metal atoms Mo1, Mo2, and Ni1 and the central amide N atoms of the dipyridylamine ligands N2, N5, N8, and N11 are disordered over two positions with a major component occupancy of 71.1(4) %. Atom pairs Mo1/Mo1A and Ni1/Ni1A were constrained to have equal anisotropic displacement parameters. The N atoms in the minor disorder component were refined with atomic displacement parameter constraints. The Mo–N distances of Mo1A were restrained to be similar.

The *para*-$CF_3$ groups of the axial $O_2CC_6H_4CF_3$ ligands are disordered over two positions, with major component occupancies of 50.02 and 59.06 % for the C48 and C56 groups, respectively. These occupancies were initially refined, then fixed to the refined value. Both disorder components of both para-$CF_3$ groups were refined with 1,2 and 1,3 geometric restraints, as well as atomic displacement parameter constraints.

The symmetry independent unit also contains three molecules of toluene, and two of the solvate molecules (corresponding to the C57 and C64 solvates) are disordered over two positions. The major component occupancy of the C64 solvate is 64.8 %. For the C57 solvate, the two disorder component occupancies are 46.9(19) and 40.6(18) %. The third molecule of toluene (corresponding to the C71 solvate) has a fractional occupancy of 92.2(9)%. The disordered solvate molecules were refined isotropically with atomic displacement parameter constraints, and all three solvate molecules were refined with idealized geometries

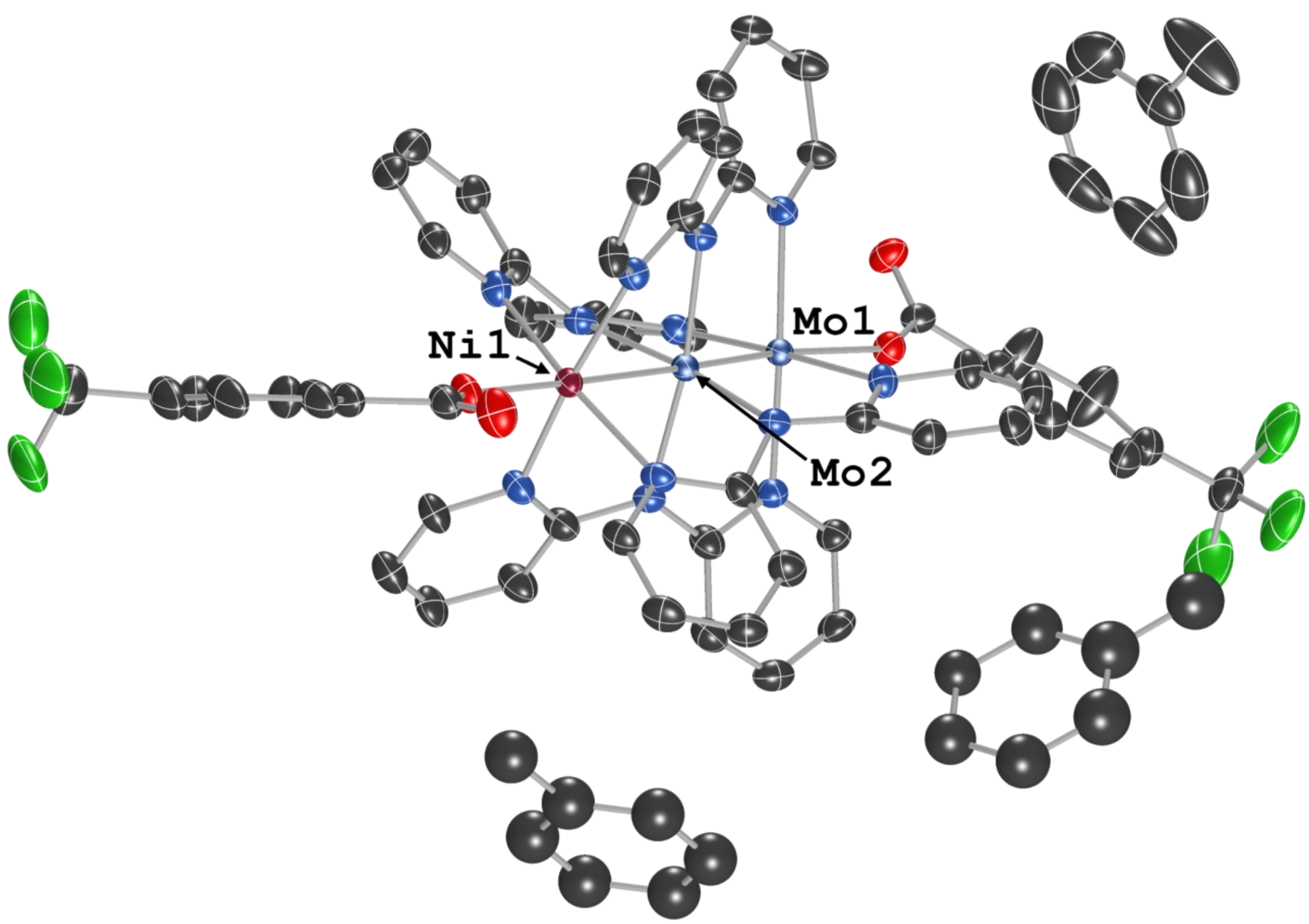


**Figure S19.** A molecular drawing of **1_CF3** shown with 50% probability ellipsoids and selected atom labels. All H atoms and minor disorder components are omitted for clarity.

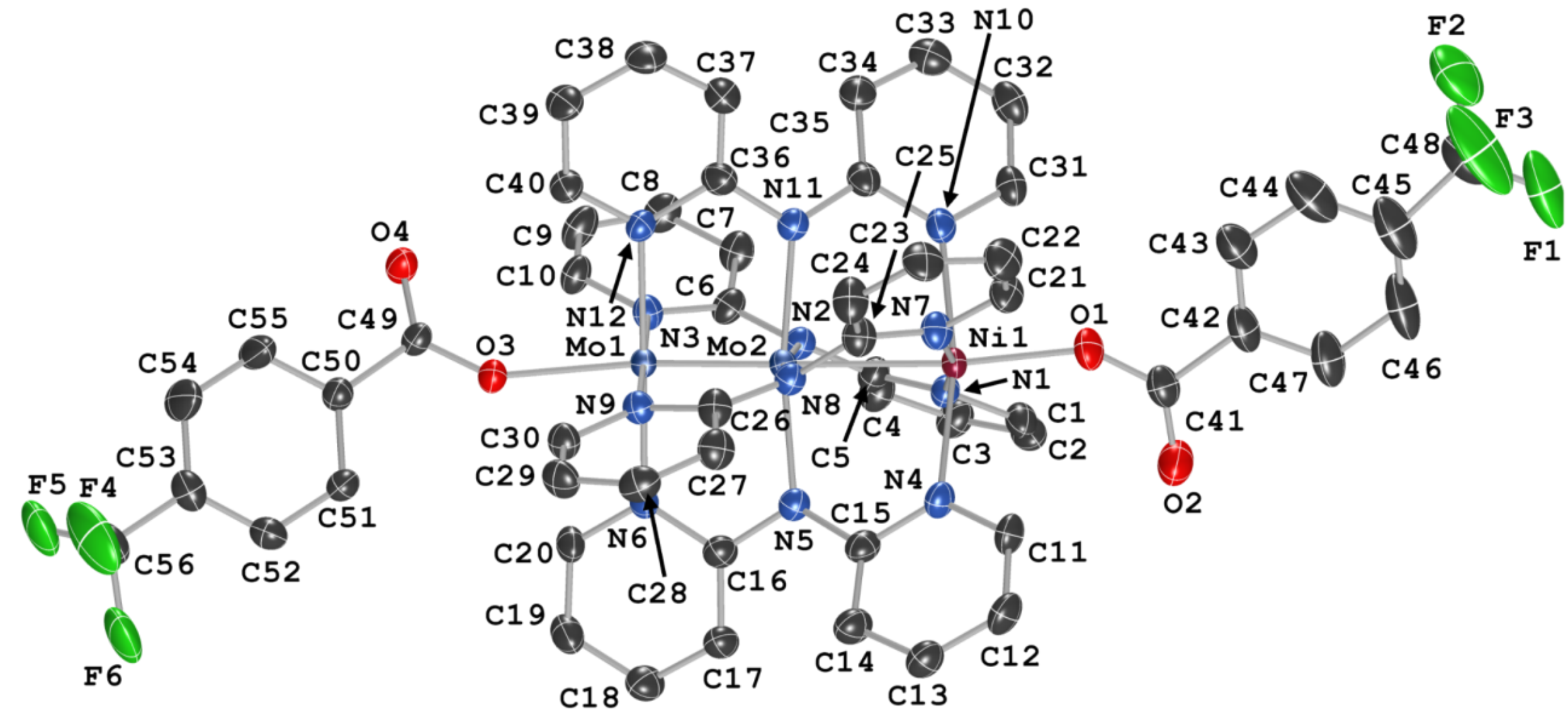


**Figure S20**. A molecular drawing of the HEMAC complex in **1_CF**. All atoms are shown with 50% probability ellipsoids; all H atoms, minor disorder components, and solvent molecules are omitted for clarity.

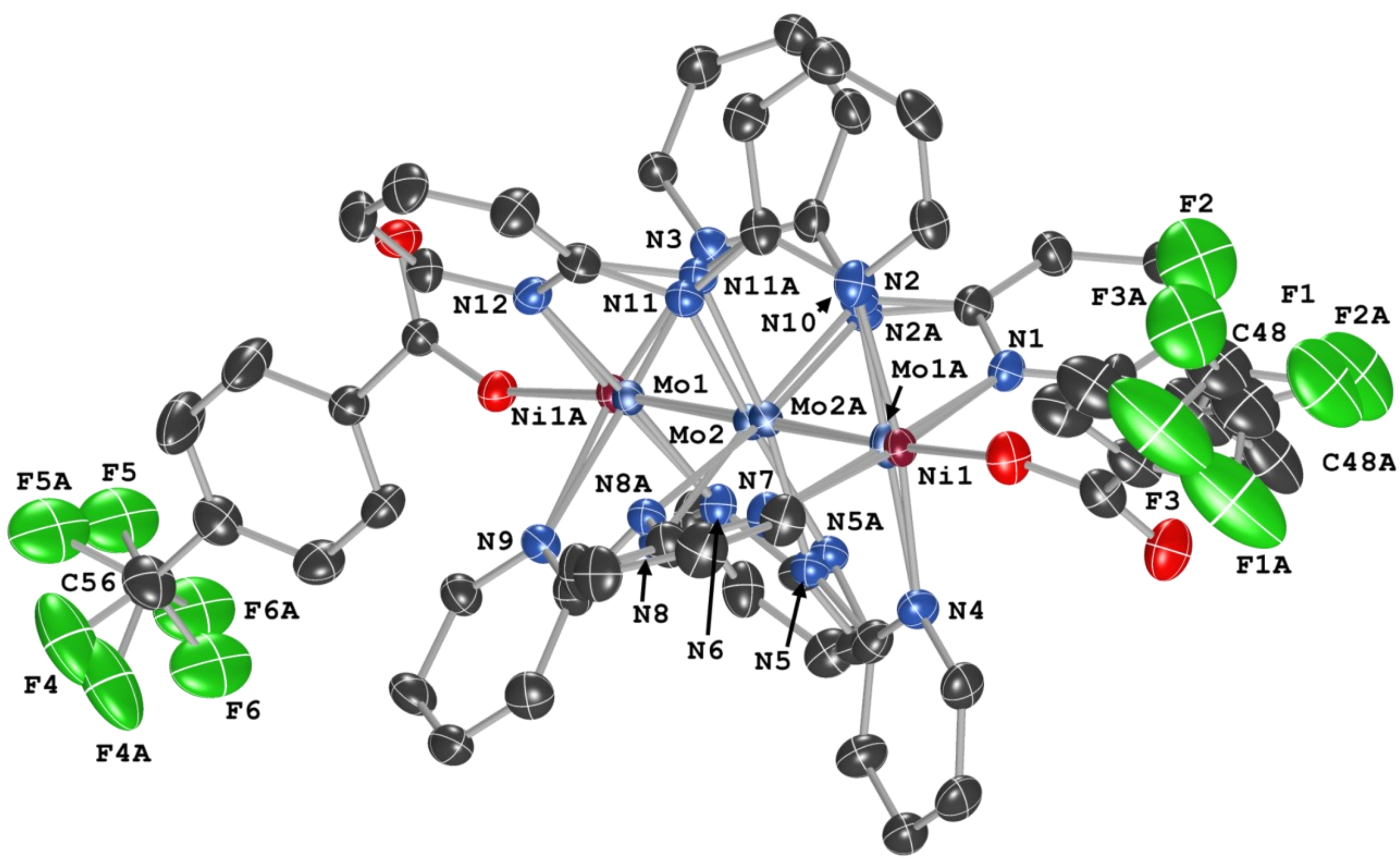


**Figure S21**. A molecular drawing of the HEMAC complex in **1_CF**$_3$ with selected atom labels and both metal atom disorder components shown. All atoms are shown with 50% probability ellipsoids; all H atoms and solvent molecules are omitted for clarity.

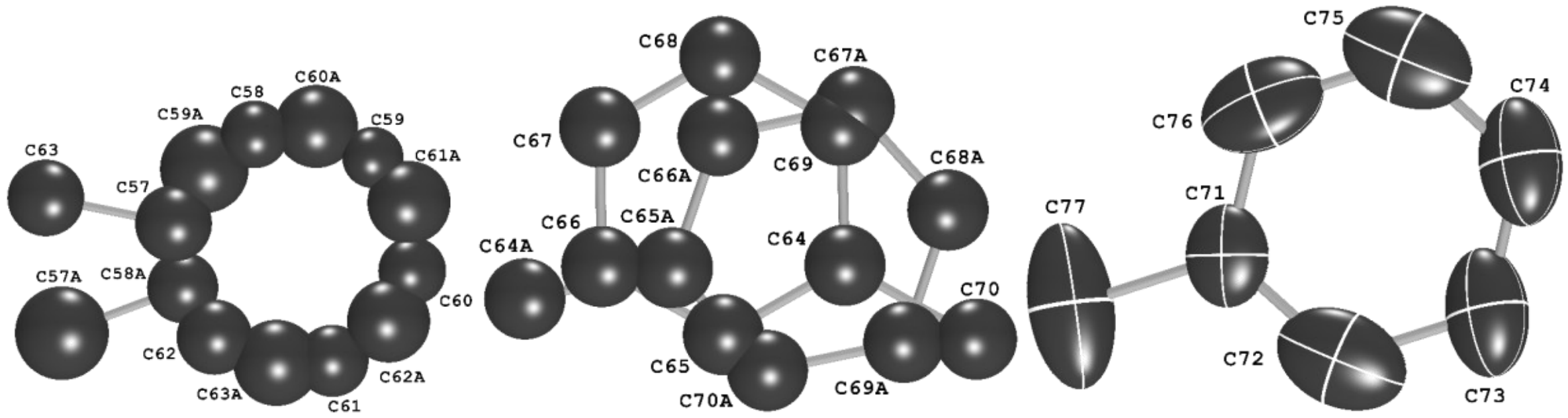


**Figure S22**. A molecular drawing of first, second, and third symmetry-independent molecules of solvate toluene in **1_CF$_3$**; all H atoms are omitted for clarity.

**4** · 2 $CH_2Cl_2$: This structure is a dichloromethane solvate of the previously reported $Mo_2Ni(dpa)_4Cl_2$ HEAMC.[6] The HEMAC complex resides on a crystallographic two-fold axis that contains the metal atoms; as such, only one half of the structure is symmetry-independent.

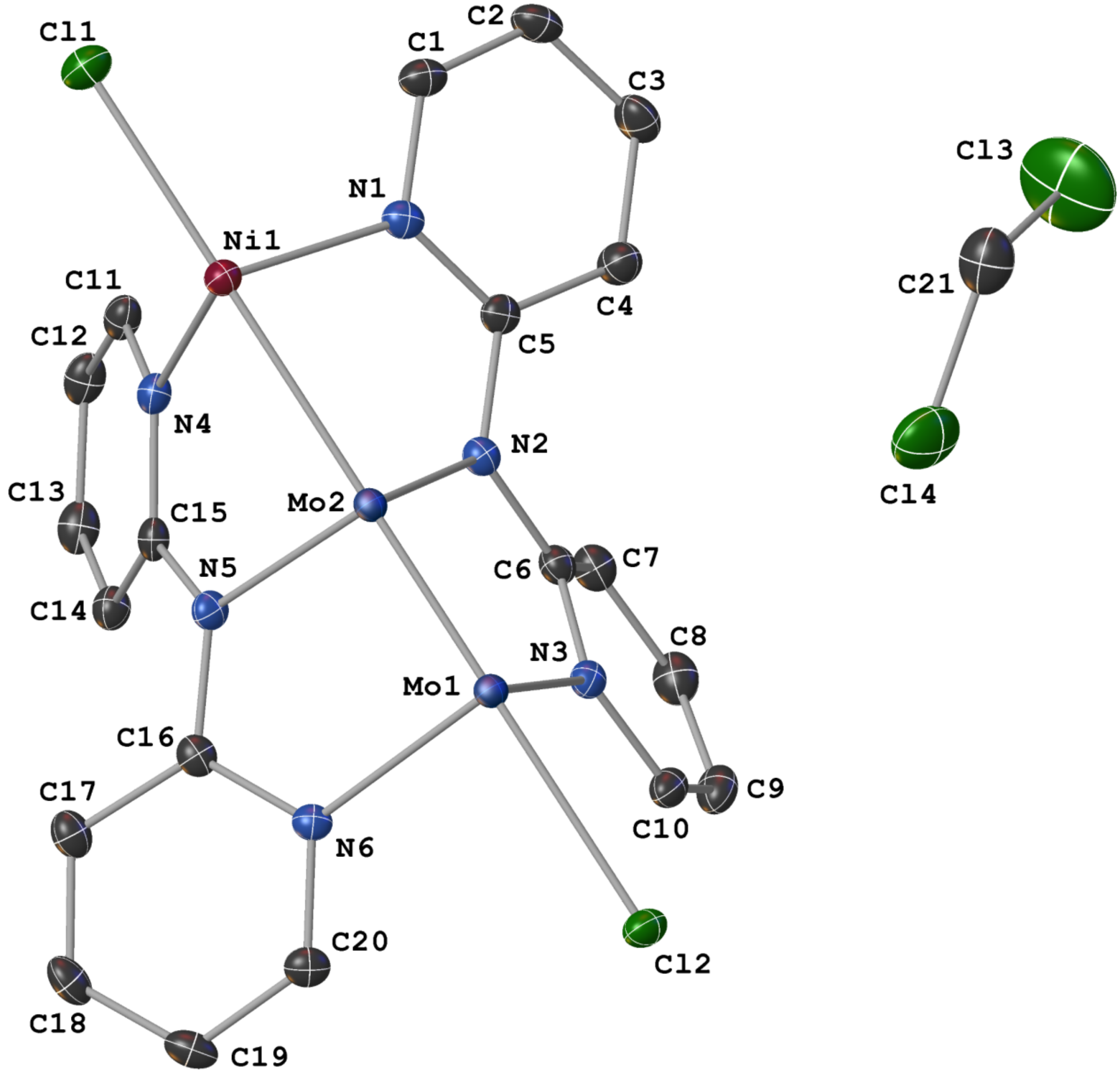


**Figure S23**: A molecular diagram of the symmetry-independent portion of $Mo_2Ni(dpa)_4Cl_2$ · 2 $CH_2Cl_2$ shown with 50 % probability ellipsoids. All H atoms are omitted.

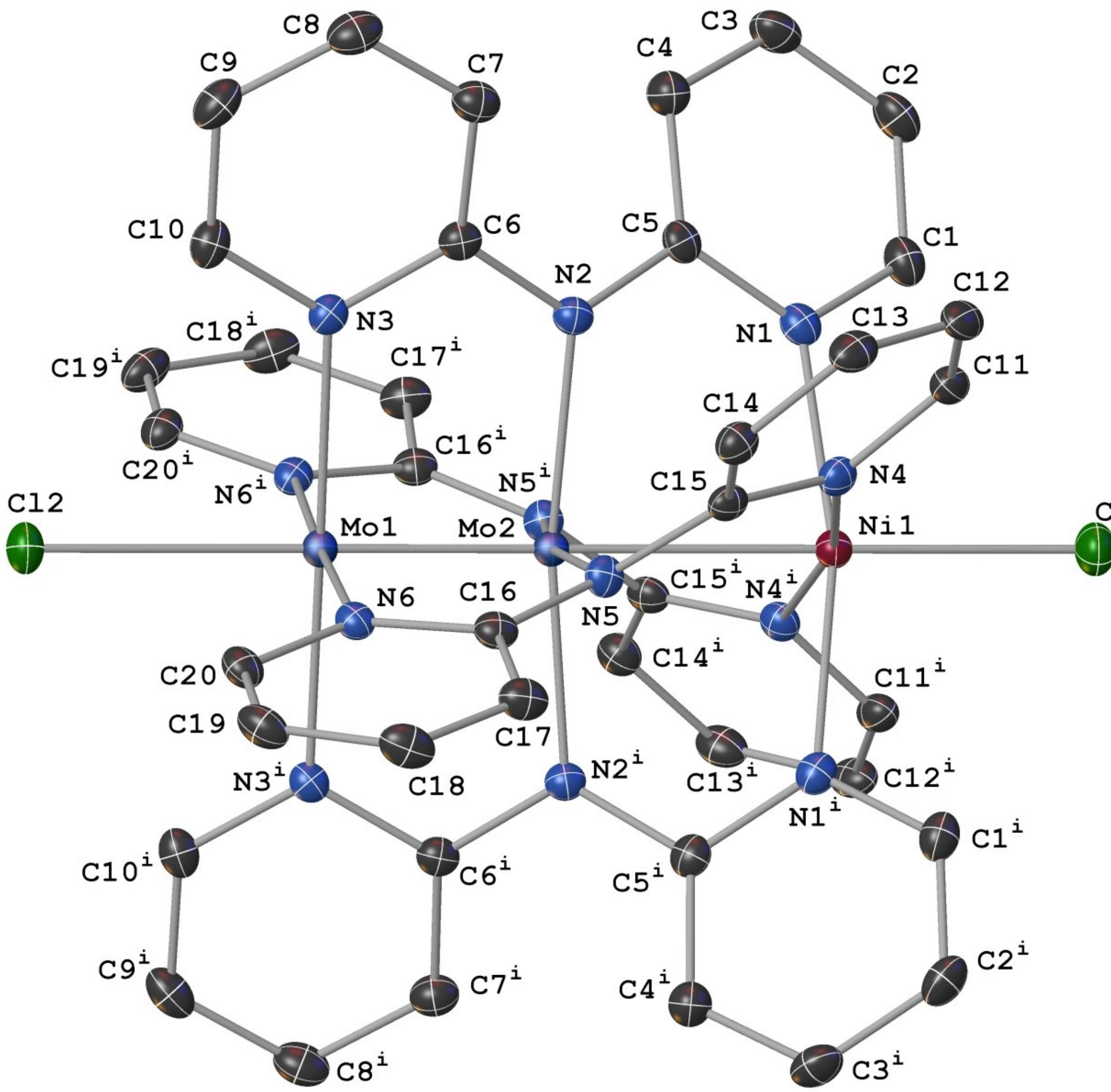


**Figure S24**: A molecular diagram of the HEMAC complex in $Mo_2Ni(dpa)_4Cl_2 \cdot 2\ CH_2Cl_2$ shown with 50 % probability ellipsoids. All H atoms and solvent molecules are omitted. [Symmetry code: (i) 1−*x*, *y*, 1/2−*z*.]

$[Mo_2(C_{10}H_8N_3)_4][O_3SCF_3]$: The structure crystallizes as a salt, with an asymmetric unit formula of $[Mo_2(C_{10}H_8N_3)_4][O_3SCF_3]$.

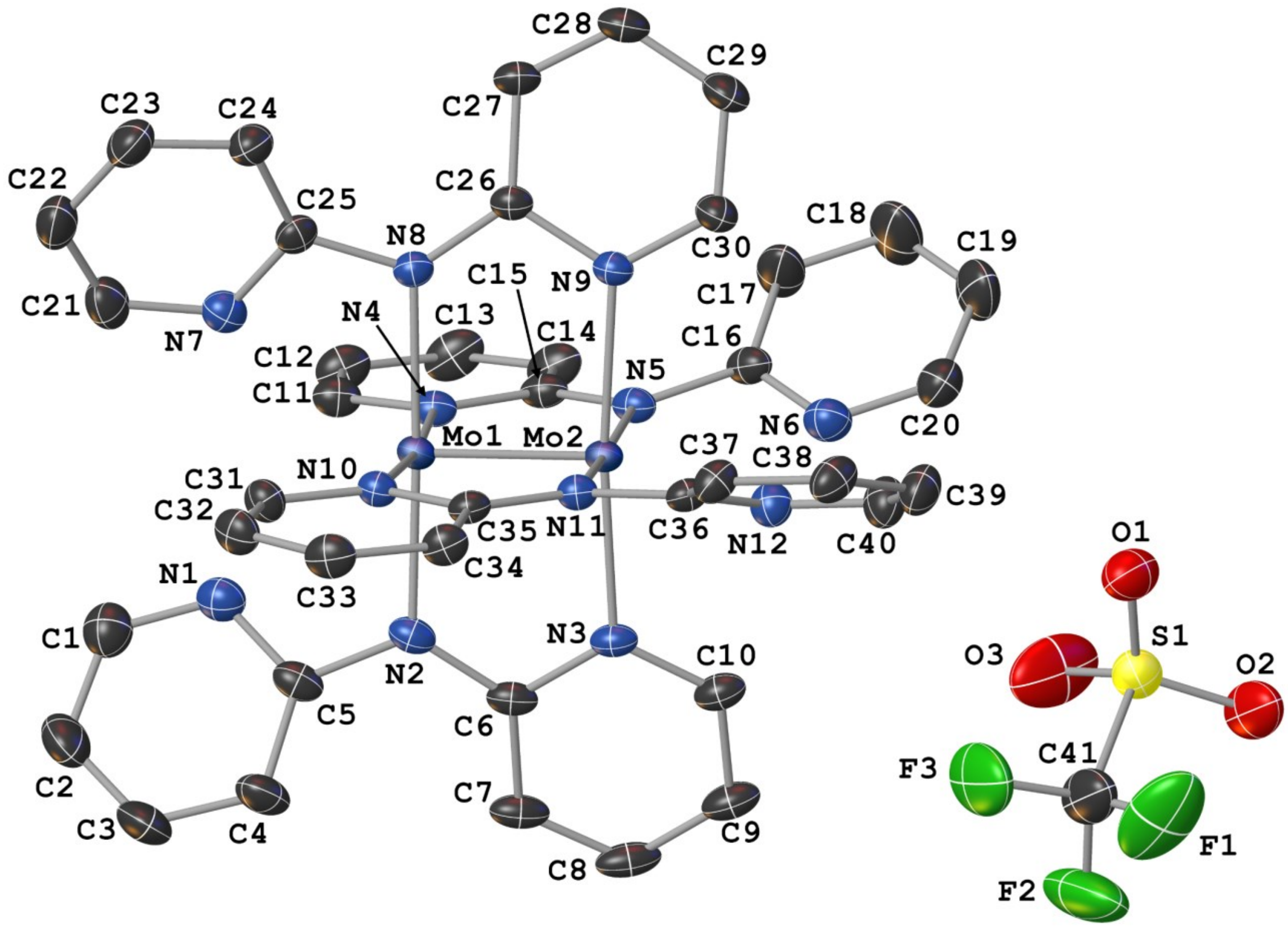


**Figure S25**. A molecular drawing of $[Mo_2(C_{10}H_8N_3)_4][O_3SCF_3]$ shown with 50% probability ellipsoids. All H atoms are omitted for clarity.

$Mo_2(C_{10}H_8N_3)_4(OAc)$: The structure crystallizes with one molecule of Mo≡Mo complex in the symmetry independent unit.

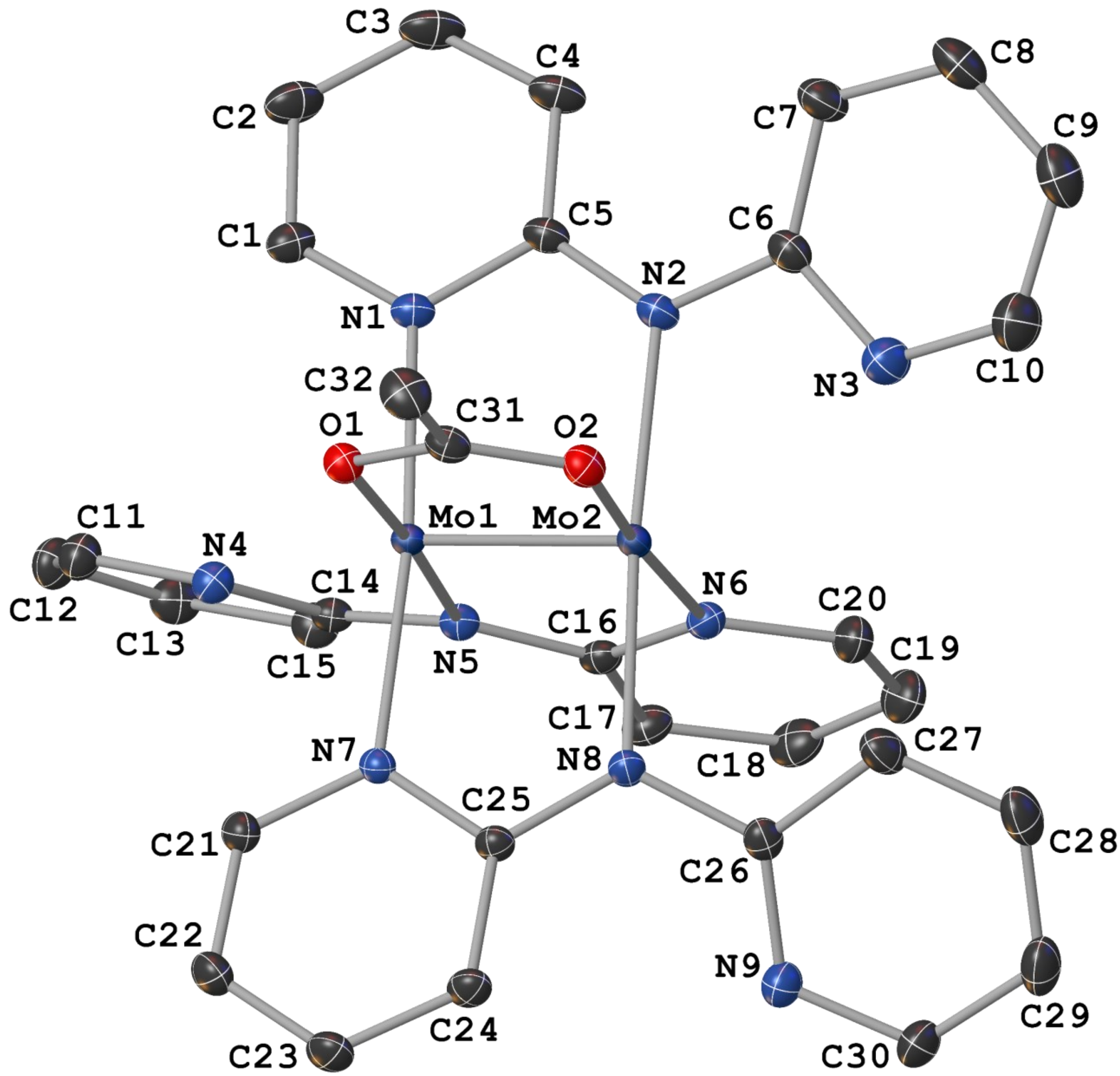


**Figure S26**. A molecular drawing of $Mo_2(dpa)_3(O_2CCH_3)$ shown with 50% probability ellipsoids. All H atoms are omitted for clarity.

**Additional comments on SQUID measurements of 1_CH$_3$, 1_H, and 1_CF$_3$.**

The $g_{\parallel}$ values for **1_CH$_3$, 1_H**, and **1_CF$_3$** are substantially smaller than those reported for the corresponding $g_{\perp}$ values for the psuedohalide series,[7] which we can attribute to differences in the fitting methods used between the series. Whereas the magnetic models for the pseudohalides were determined from susceptibility measurements alone, the models provided here were determined from simultaneous fits of the susceptibility and the reduced magnetization data. Fits of the susceptibility data for **1_CH$_3$, 1_H**, and **1_CF$_3$** independent of the reduced magnetization data provided models with $g_{\parallel}$ values of 2.000(3) for **1_CH$_3$**, 2.189(4) for **1_H**, and 2.499(9) for **1_CF$_3$** similar to those seen with the pseudohalides. While these alternative fits are reported in **Table S4** we believe that the magnetic parameters derived from the simultaneous fit provide a better reflection of the true behavior of the system.

**Table S3**. SQUID fit parameters for **1** and **3** from models only fitting susceptibility data

| | $g_{\parallel}$ | $g_{\perp}$ | D ($cm^{-1}$) | TIP | Residual |
|---|---|---|---|---|---|
| **1_$CH_3$** | 2.000(3) | 1.898(2) | 7.40(2) | 0.002649(3) | 0.000204 |
| **1_H** | 2.189(4) | 1.877(2) | 8.41(2) | 0.002399(3) | 0.000237 |
| **1_$CF_3$** | 2.499(9) | 1.895(7) | 6.51(10) | 0.014768(4) | 0.0391 |

Residual = $\left[\sum_{i=1}^{points}\left(\chi_{exp}-\chi_{calc}\right)^2\right]$ where $\chi_{exp}/\chi_{calc}$ = measured/calculated susceptibility

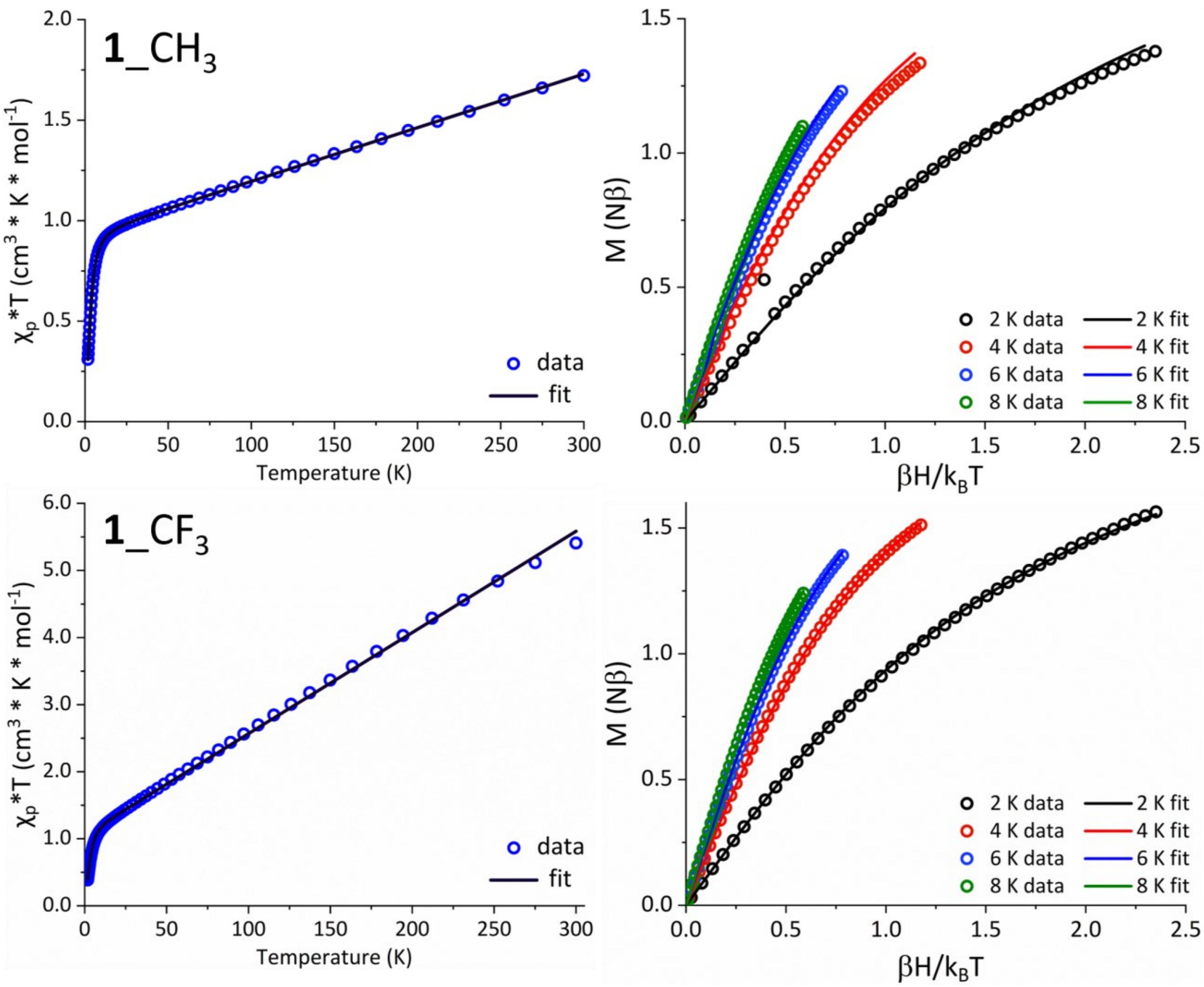


**Figure S27.** The magnetic susceptibility (left) and reduced magnetization (right) data and fits for **1_$CH_3$** and **1_$CF_3$**. Note: The outlier data point of the 2 K isotherm of **1_$CH_3$** was omitted from the fit.

**Table S4**. Electrochemical data for compound 2

| $Mo_2^{4+}/Mo_2^{5+}$ | | | | | Fc/Fc$^+$ | | | | |
|---|---|---|---|---|---|---|---|---|---|
| $E_{1/2}$ (V) | $\Delta E_p$ (mV) | $I_{pc}$ (mA) | $I_{pa}$ (mA) | $I_{pc}/I_{pc}$ | $E_{1/2}$ (V) | $\Delta E_p$ (mV) | $I_{pc}$ (mA) | $I_{pa}$ (mA) | $I_{pc}/I_{pc}$ |
| -0.324 | 65 | 0.00102 | 0.00125 | 0.816 | 0.138 | 113 | 0.00227 | 0.00271 | 0.838 |

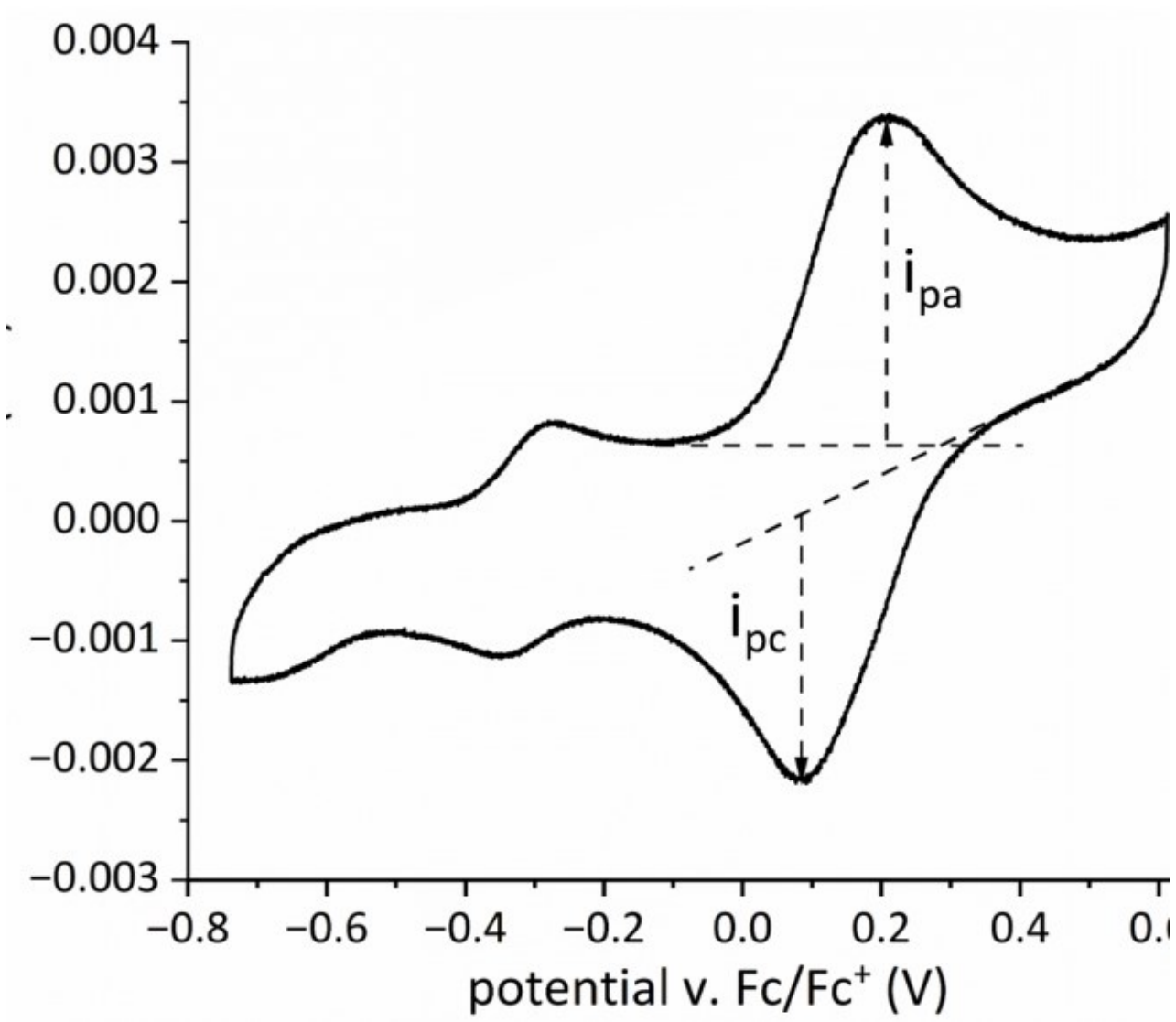


**Figure S28**. The cyclic voltammogram of compound **2** showing the peak height analysis for the axial $O_2CFc^0$/ $O_2CFc^+$ couple.

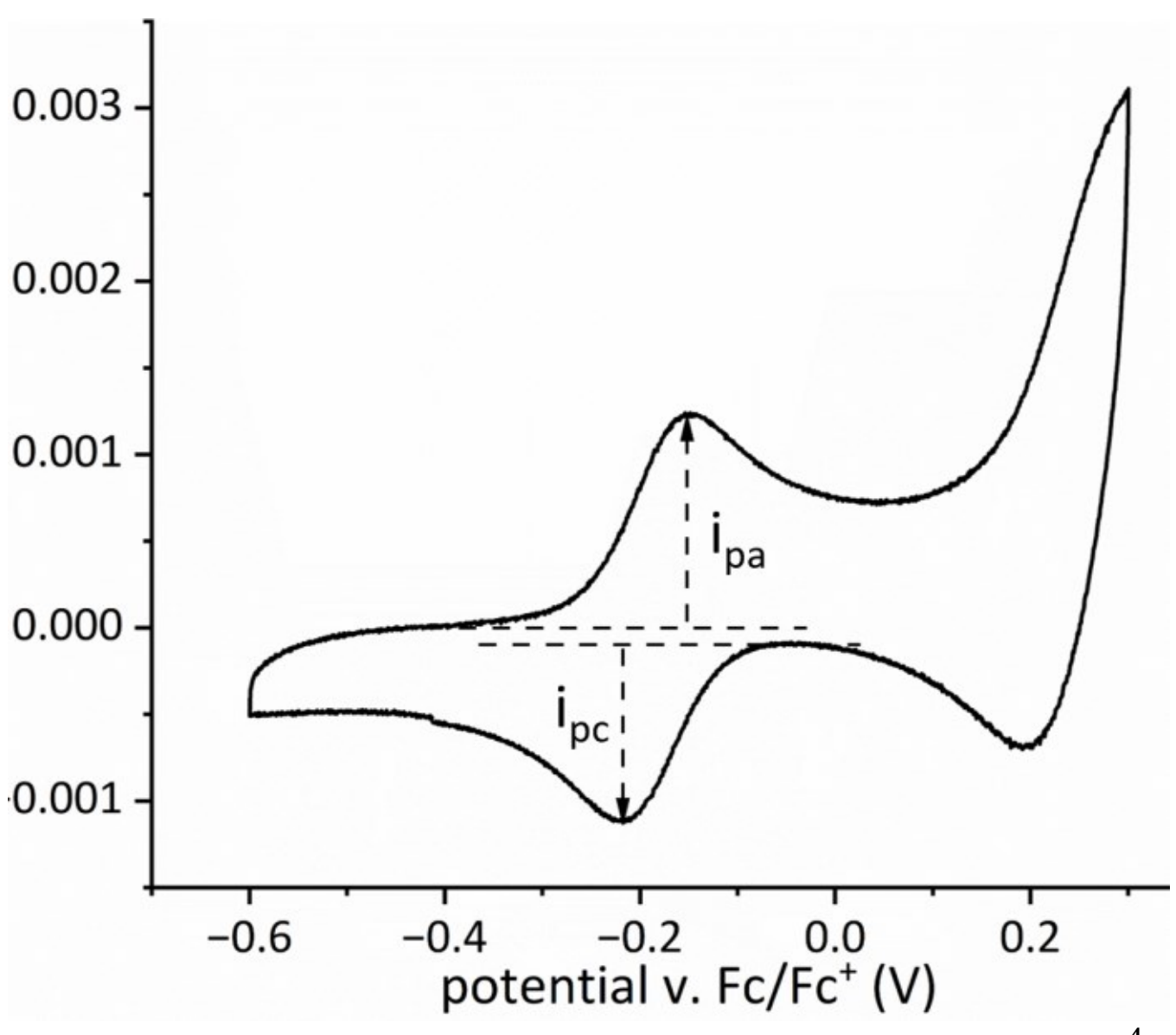


**Figure S29**. The cyclic voltammogram of the $Mo_2^{4+}/Mo_2^{5+}$ couple for compound **2** showing the peak height analysis.

**References**


(1) *APEX3 : Suite for Crystallographic Software. Single Crystal*; Bruker Nano Inc: Madison WI, 2019.

(2) Sheldrick, G. SHELXT - Integrated space-group and crystal-structure determination. *Acta. Crystallogr. Sect. A* **2015**, *71* (1), 3-8. DOI: 10.1107/S2053273314026370

(3) Dolomanov, O. V.; Bourhis, L. J.; Gildea, R. J.; Howard, J. A. K.; Puschmann, H. OLEX2: a complete structure solution, refinement and analysis program. *J. Appl. Crystallogr.* **2009**, *42* (2), 339-341. DOI: 10.1107/S0021889808042726

(4) Spek, A. L. PLATON SQUEEZE: a tool for the calculation of the disordered solvent contribution to the calculated structure factors. *Acta Crystallogr. Sect. C* **2015**, *71* (1), 9-18. DOI: 10.1107/s2053229614024929

(5) Guzei, I. A. An idealized molecular geometry library for refinement of poorly behaved molecular fragments with constraints. *J. Appl. Crystallogr.* **2014**, *47* (2), 806-809. DOI: 10.1107/s1600576714004427

(6) Chipman, J. A.; Berry, J. F. Extraordinarily Large Ferromagnetic Coupling ($J \geq 150$ $cm^{-1}$ ) by Electron Delocalization in a Heterometallic Mo≣Mo–Ni Chain Complex. *Chem. Eur. J.* **2018**, *24* (7), 1494-1499. DOI: 10.1002/chem.201704588

(7) Chipman, J. A.; Berry, J. F. Facile Axial Ligand Substitution in Linear Mo≣Mo-Ni Complexes. *Inorg. Chem.* **2018**, *57* (15), 9354-9363. DOI: 10.1021/acs.inorgchem.8b01331
